# GWAS on Imputed Whole-Genome Resequencing From Genotyping-by-Sequencing Data for Farrowing Interval of Different Parities in Pigs

**DOI:** 10.3389/fgene.2019.01012

**Published:** 2019-10-18

**Authors:** Pingxian Wu, Kai Wang, Jie Zhou, Dejuan Chen, Qiang Yang, Xidi Yang, Yihui Liu, Bo Feng, Anan Jiang, Linyuan Shen, Weihang Xiao, Yanzhi Jiang, Li Zhu, Yangshuang Zeng, Xu Xu, Xuewei Li, Guoqing Tang

**Affiliations:** ^1^Farm Animal Genetic Resources Exploration and Innovation, Key Laboratory of Sichuan Province, Sichuan Agricultural University, Chengdu, China; ^2^Sichuan Province Department of Agriculture and Rural Affairs, Sichuan Animal Husbandry Station, Chengdu, China; ^3^College of Life Science, Sichuan Agricultural University, Yaan, China

**Keywords:** imputation, genome-wide association study, genotyping-by-sequencing, resequencing, farrowing interval, pigs

## Abstract

The whole-genome sequencing (WGS) data can potentially discover all genetic variants. Studies have shown the power of WGS for genome-wide association study (GWAS) lies in the ability to identify quantitative trait loci and nucleotides (QTNs). However, the resequencing of thousands of target individuals is expensive. Genotype imputation is a powerful approach for WGS and to identify causal mutations. This study aimed to evaluate the imputation accuracy from genotyping-by-sequencing (GBS) to WGS in two pig breeds using a resequencing reference population and to detect single-nucleotide polymorphisms (SNPs) and candidate genes for farrowing interval (FI) of different parities using the data before and after imputation for GWAS. Six hundred target pigs, 300 Landrace and 300 Large White pigs, were genotyped by GBS, and 60 reference pigs, 20 Landrace and 40 Large White pigs, were sequenced by whole-genome resequencing. Imputation for pigs was conducted using Beagle software. The average imputation accuracy (allelic *R* ^2^) from GBS to WGS was 0.42 for Landrace pigs and 0.45 for Large White pigs. For Landrace pigs (Large White pigs), 4,514,934 (5,533,290) SNPs had an accuracy >0.3, resulting an average accuracy of 0.73 (0.72), and 2,093,778 (2,468,645) SNPs had an accuracy >0.8, resulting an average accuracy of 0.94 (0.93). Association studies with data before and after imputation were performed for FI of different parities in two populations. Before imputation, 18 and 128 significant SNPs were detected for FI in Landrace and Large White pigs, respectively. After imputation, 125 and 27 significant SNPs were identified for dataset with an accuracy >0.3 and 0.8 in Large White pigs, and 113 and 18 SNPs were found among imputed sequence variants. Among these significant SNPs, six top SNPs were detected in both GBS data and imputed WGS data, namely, SSC2: 136127645, SSC5: 103426443, SSC6: 27811226, SSC10: 3609429, SSC14: 15199253, and SSC15: 150297519. Overall, many candidate genes could be involved in FI of different parities in pigs. Although imputation from GBS to WGS data resulted in a low imputation accuracy, association analyses with imputed WGS data were optimized to detect QTNs for complex trait. The obtained results provide new insight into genotype imputation, genetic architecture, and candidate genes for FI of different parities in Landrace and Large White pigs.

## Introduction

Reproductive traits play an important role in pig industry and directly affect the sow reproductive performance. In recent years, the researchers extensively studied reproductive traits (such as litter size, birth weight, and number of teats) and identified many quantitative trait loci (QTLs) and candidate genes in pigs. However, a few studies focused on farrowing interval (FI). FI was defined as the number of days between two consecutive litters in sow’s productive life. This trait is one of the major determinants of the efficiency of sow reproduction. The heritability ranged from 0.04 to 0.16 for FI in pigs (Serenius et al., 2003; Cavalcante Neto et al., 2009). The previous literatures have shown that these traits in different parities should be considered as different traits for farrowing traits (Roehe and Kennedy, 1995; Noguera et al., 2002; Onteru et al., 2011). Thus, FIs of different parities need to be used as different traits in analyses. In summary, a total of 28,720 QTLs were reported for 677 complex traits in pigs (PigQTL, https://www.animalgenome.org/cgi-bin/QTLdb/SS/index, March 1, 2019). Among them, 2,129 QTLs were associated with reproductive traits. However, there are no QTLs and genes found for FI in pigs.

Genome-wide association studies (GWASs) are an effective method for identifying the genetic variations involved in complex traits. In pigs, GWAS has been widely conducted to uncover the genetic architecture behind economically important traits, such as reproduction- (Wang et al., 2018), growth-, and meat-related traits (Jiang et al., 2018). Using this approach, a range of quantitative trait nucleotides, quantitative trait genes, and QTLs involved in important traits in pigs were identified (Jun et al., 2011; Ma et al., 2014; Derks et al., 2017; Zhang et al., 2018). However, because of the limited number of single-nucleotide polymorphisms (SNPs), the power of GWAS is very limited, and several causal loci were missed in previous studies. This led to an inability to identify the causal loci of complex traits. Whole-genome sequence (WGS) data containing the majority of SNPs were optimized to enhance the accuracy and power of GWAS and the detection of QTLs associated with complex traits. To obtain credible GWAS results, a large number of genotyped individuals were required in association analyses. Although the cost of resequencing is rapidly decreasing, it is still expensive to resequence thousands of individuals. An efficient imputation strategy for WGS data was thus recommended to detect causal loci. Using this method, low-density SNPs were imputed to high-density or WGS data at low cost. A small number of resequenced individuals (called as “reference population”) and a large number of individuals with low-density SNPs (called as “target population”) were used for genotype imputation. Based on the reference and target genotype data, genotype imputation used linkage disequilibrium (LD) of haplotypes in reference sequence data to predict the SNPs missing from target sequence data. Then, low-density SNPs were imputed to WGS data using the reference data.

Recently, genotype imputation has been successfully implemented and obtained reliable results in humans (Howie et al., 2012) and livestock (Sanchez et al., 2017; Ye et al., 2018; Berg et al., 2019). In cattle, 12 QTLs for mammary gland morphology (Pausch et al., 2016) and 34 QTLs for milk protein composition (Sanchez et al., 2017) were found using imputed data. Based on the imputed WGS data, Berg et al. (2019) reported that the detected QTLs increased with increasing SNP density and identified a clear peak on SSC7 for teats number in Large White and Dutch Landrace pigs. To detect the missing QTLs, the imputed WGS data were used to perform association analyses, and an important QTL was detected on SSC1 for lumbar number in Sutai pigs (Yan et al., 2017).

In general, the imputed data contributed benefit for association studies. However, only few literatures reported the factors that affected genotype imputation in livestock. According to the reported literatures, the imputation accuracy was affected by sequencing depth, size of reference population, the relationship between reference and target population (Ye et al., 2018), and marker density of target population (Ventura et al., 2016). Because the imputation would result in a poor imputation accuracy using multiple reference populations (Berg et al., 2019), a single-breed reference population may be optimal for imputation. To date, few studies analyzed the imputation accuracy from real genotype to imputed WGS data and performed GWAS using imputed WGS data in livestock.

To the best of our knowledge, no studies have reported on the imputation of genotyping-by-sequencing (GBS) data to WGS using whole-genome resequencing data of individuals as a reference population. In this study, the target populations were genotyped by GBS technology, and the reference populations were sequenced using whole-genome resequencing. Then, association analyses were performed for both the unimputed and imputed data. In this context, the objectives of this study were (1) to impute the GBS data to WGS data and analyze the accuracy of imputation to WGS and (2) to perform GWAS to reveal the genetic architecture behind FI of different parities in Large White and Landrace pigs.

## Methods

### Animals and Phenotype Records

A total of 660 pigs, 320 Landrace and 340 Large White pigs, from the national core pig breeding farm of Sichuan Tianzow Breeding Technology Co., Ltd. (http://www.tianzow.com/areashow.php?id=790, Nanchong, China), were used in this study. The ear tissues for 660 pigs were collected and stored in 75% alcohol, which was approved by the Institutional Animal Care and Use Committee of the Sichuan Agricultural University (DKY-B20140302).

All pigs with common genetic background were introduced from Canadian Hylife Company at 2008. The farrowing records were collected from parity 1 to 4 during the period of 2012–2015. The FI values were defined as the number of days between two adjacent litters. Due to the different genetic architecture of each parity, FIs of different parities were considered as different traits. The following reproductive traits were defined and recorded for each pig: (1) FI from parity 1 to 2 (FI_L12), (2) parity 2 to 3 (FI_L23), and (3) parity 3 to 4 (FI_L34) in Landrace pigs; and (4) FI from parity 1 to 2 (FI_Y12), (5) parity 2 to 3 (FI_Y23), and (6) parity 3 to 4 (FI_Y34) in Large White pigs. Totals of 1,980 FI records were collected. The normal transformation of phenotypic data was conducted by R software (Aulchenko et al., 2007).

### DNA Extraction

The genomic DNA was extracted from ear tissues using the OMEGA Tissue DNA Kit (Omega Bio-Tek) as per the manufacturer's instructions. The Nanodrop-2000 spectrophotometer was used to measure the quality and quantity of the genomic DNA samples. The genomic DNA samples with the ratio of light absorption (A260/280) between 1.8 and 2.0, concentration ≥50 ng/µL, and total volume ≤50 µL were used for sequencing.

### Reference Sequence Data

A total of 60 pigs, 20 Landrace and 40 Large White pigs, were selected for resequencing by random selection. The resequencing was performed by Illumina HiSeq PE150 platform, with average sequencing depth of 20-fold. The initial quality of resequencing data was performed by FastQC (http://www.bioinformatics.bbsrc.ac.uk/projects/fastqc/); 3.0-T clean data were contained. The clean reads were mapped to Sscrofa11.1 reference sequence by the BWA (version 0.7.15) software (Li and Durbin, 2009). After that, GATK (version 3.5) software (Depristo et al., 2011) was used to realign the mapped reads and called SNPs. A total of 21,104,245 SNPs were called by GATK. A quality control procedure was adopted by removing SNPs with minor allele frequency (MAF) <0.05, missing rate (Miss) >0.1, Hardy–Weinberg equilibrium (HWE) >1.0 × 10^-6^, read depth (dp) <6, and SNPs with no position information and located on sex chromosomes. Quality control was conducted using VCFtools (version 4.2) (Danecek et al., 2011). After quality control, a total of 10,501,384 SNPs remained. The WGS data were used as the reference sequence for further study.

### Target Sequence Data

The remained 600 pigs, 300 Landrace and 300 Large White pigs, were selected as the target population. A total of 600 samples were genotyped using GBS with Illumina HiSeq PE150 platform. Quality control with MAF >0.01, Miss <0.2, HWE <1.0 × 10^-6^, and dp > 3 was performed by VCFtools (version 4.2) (Danecek et al., 2011). Then, the SNPs with no position information and located on sex chromosomes were excluded from this dataset. After quality control, a total of 325,557 SNPs were retained for the target population.

### Genotype Imputation

The imputation from GBS SNP genotypes to WGSs for Large White pigs and Landrace pigs was performed by Beagle (version 3.3.2) (Browning and Browning, 2007) with default parameter settings. The genotype imputation was separately conducted for each breed. For Landrace pigs, the GBS data for 300 target Landrace pigs were imputed to WGS data, using the WGS reference data of 20 Landrace pigs. For Large White pigs, using the WGS reference data of 40 Large White pigs, the GBS data of 300 target Large White pigs were imputed to WGS data. The imputation accuracy at each SNP was assessed using the estimated squared correlation between the allele dosage and true allele dosage for the marker (allelic *R* ^2^). After imputation, two filter criteria were conducted: (1) removing SNPs with an imputation accuracy < 0.3 and MAF < 0.01 and (2) removing SNPs with an imputation accuracy < 0.8 and MAF < 0.01.

### Genome-Wide Association Study

Single marker regression analyses were performed independently on GBS data and imputed genotype data using GEMMA (Depristo et al., 2011) software. The following univariate mixed liner model was used to test the association between SNPs and FI:

y=Xα+Zβ+Wa+e

where **y** is the vector of phenotypic values; **α** is the vector of fixed effects, including farrowing year, farrowing month, parity; **β** is the marker effects; **a** is the vector of the remaining polygene effect; **e** is the vector of residual effects; **X**, **Z**, and **W** are incidence matrices for **α**, **β**, and **a**, respectively. The Bonferroni correction method was used to determine the threshold values in this study. The genome-wide significance level (0.05/*N*) and suggestive level (1/*N*) were used in this study, where *N* is the number of analyzed SNPs (**Supplementary Table 1**).

The Manhattan and QQ plots were drawn by R package “qqman” (Turner, 2014). The genomic inflation factor (λ = the observed *P* value/the expected *P* values) was calculated to evaluate the extent of false positive signals using the GenABEL package in R (Aulchenko et al., 2007).

### Candidate Genes Searching

In order to highlight candidate genes at genome-wide significant loci, candidate genes were searched within a 20-Kb region centering each top SNP on pig genes Sscrofa11.1 (http://asia.ensembl.org/biomart/martview/). The gene function was carried out by NCBI database (https://www.ncbi.nlm.nih.gov/) based on the description of gene function and reported literatures. Furthermore, this study performed the gene ontology (GO) analyses by DAVID Bioinformatics Resources (Dennis et al., 2003). The Fisher exact test was used to detect the significant GO terms, and the genes involved in significant GO terms (*P* < 0.05) were used for further analyses (Dennis et al., 2003; Rivals et al., 2007).

## Results

### Genotype Data

The overview of numbers of SNPs for each breed is shown in **Table 1**. After quality control, a total of 10,501,384 and 325,557 SNPs remained from 18 autosomes for reference and target population, respectively. After imputation, this study obtained 12,835,977 and 13,144,579 SNPs from 18 autosomes for 300 Landrace and 300 Large White pigs, respectively. After removing SNPs with allelic *R* ^2^ < 0.3, a total of 4,514,934 SNPs for Landrace pigs and 5,533,290 SNPs for Large White pigs were retained. After filtering SNPs with allelic *R* ^2^ <0.8, 2,093,778 and 2,468,645 were retained for Landrace and Large White pigs, respectively.

**Table 1:** Number of SNPs before and after imputation with different filterings from GBS to WGS data.

Breed	Chromosome	Before imputation	After imputation		
			Before filtering	After filtering (*R* ^2^ >0.3)	After filtering (*R* ^2^ >0.8)
Landrace pig	1	33,648	1,163,674	411,803	200,693
2	23,549	1,056,375	348,556	151,185	
3	19,115	741,850	283,255	132,967	
4	19,438	677,120	270,867	131,240	
5	15,492	613,081	184,337	84,815	
6	19,724	821,252	285,405	129,414	
7	19,812	724,764	268,144	123,498	
8	20,814	784,755	287,282	128,842	
9	20,455	856,362	298,509	128,612	
10	15,627	633,875	226,590	103,204	
11	13,703	491,636	180,753	84,277	
12	9,776	443,666	133,030	56,870	
13	24,232	924,908	332,499	155,654	
14	19,558	859,867	307,671	157,568	
15	17,278	672170	211,950	97,611	
16	13,863	551,359	215,034	105v006	
17	10,879	486,742	138,882	58,702	
18	8,594	332,521	130,367	63,620	
Large White pig	1	33,648	1,258,467	575,618	274,460
2	23,549	1,001,874	362,988	151,533	
3	19,115	755,528	304,432	131,781	
4	19,438	693,446	341,445	166,814	
5	15,492	627,469	260,109	115,518	
6	19,724	884,061	337,922	139,947	
7	19,812	785,970	304,112	130,640	
8	20,814	803,550	352,306	154,089	
9	20,455	782,655	351,867	162,192	
10	15,627	669,065	242,483	93,945	
11	13,703	579,287	242,877	102,102	
12	9,776	465,664	166,687	67,556	
13	24,232	1,007,593	461,286	226,507	
14	19,558	836,804	385,503	185,524	
15	17,278	658,978	289,207	132,518	
16	13,863	540,903	225,310	90,049	
17	10,879	467,065	182,444	75,628	
18	8,594	326,200	146,694	67,842	

### Imputation Accuracy

**Figure 1:**
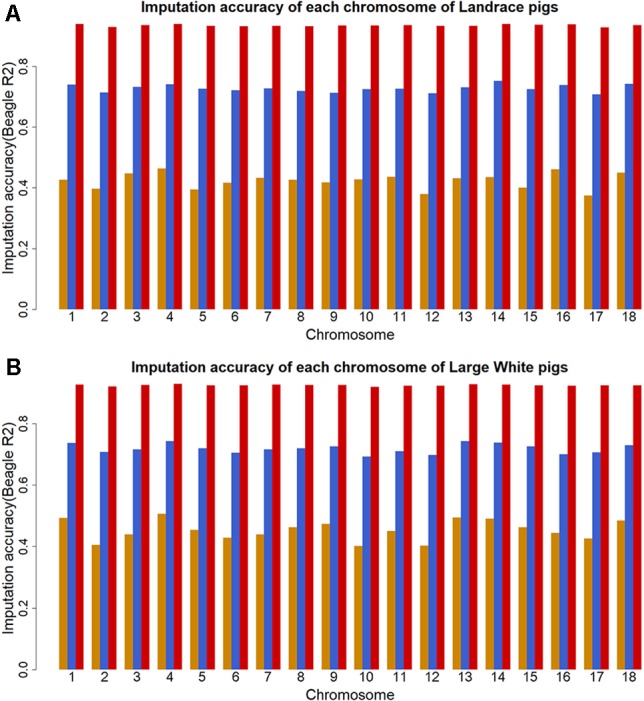
Imputation accuracy from GBS to WGS data for each chromosome in Landrace **(A)** and Large White pigs **(B)**. Imputation accuracy before filtering (orange), after filtering with allelic *R*^2^ > 0.3 (blue), and after filtering with allelic *R*^2^ > 0.8 (red).

The genotype imputation was conducted by Beagle software, and imputation accuracy at each SNP was assessed using allelic *R* ^2^. The plots of imputation accuracy are shown in **Figure 1** for each chromosome before and after filtering data. After imputation, the GBS data were imputed to WGS data with a poor imputation accuracy. The average accuracy of whole genome was 0.42 for Landrace pigs and 0.45 for Large White pigs. The lowest accuracy and highest accuracy were 0.37 (for SSC17) and 0.46 (for SSC4) in Landrace pigs. For Large White pigs, the lowest accuracy and highest accuracy were 0.40 (for SSC10) and 0.51 (for SSC4). After quality control, these retained SNPs had accuracy lower than 0.3, resulting in an average accuracy of 0.73 and 0.72 for Landrace and Large White pigs. After removing the SNPs with the accuracy lower than 0.8, the average accuracies were 0.94 and 0.93 for Landrace and Large White pigs.

### Factors That Affect Imputation Accuracy

**Figure 2:**
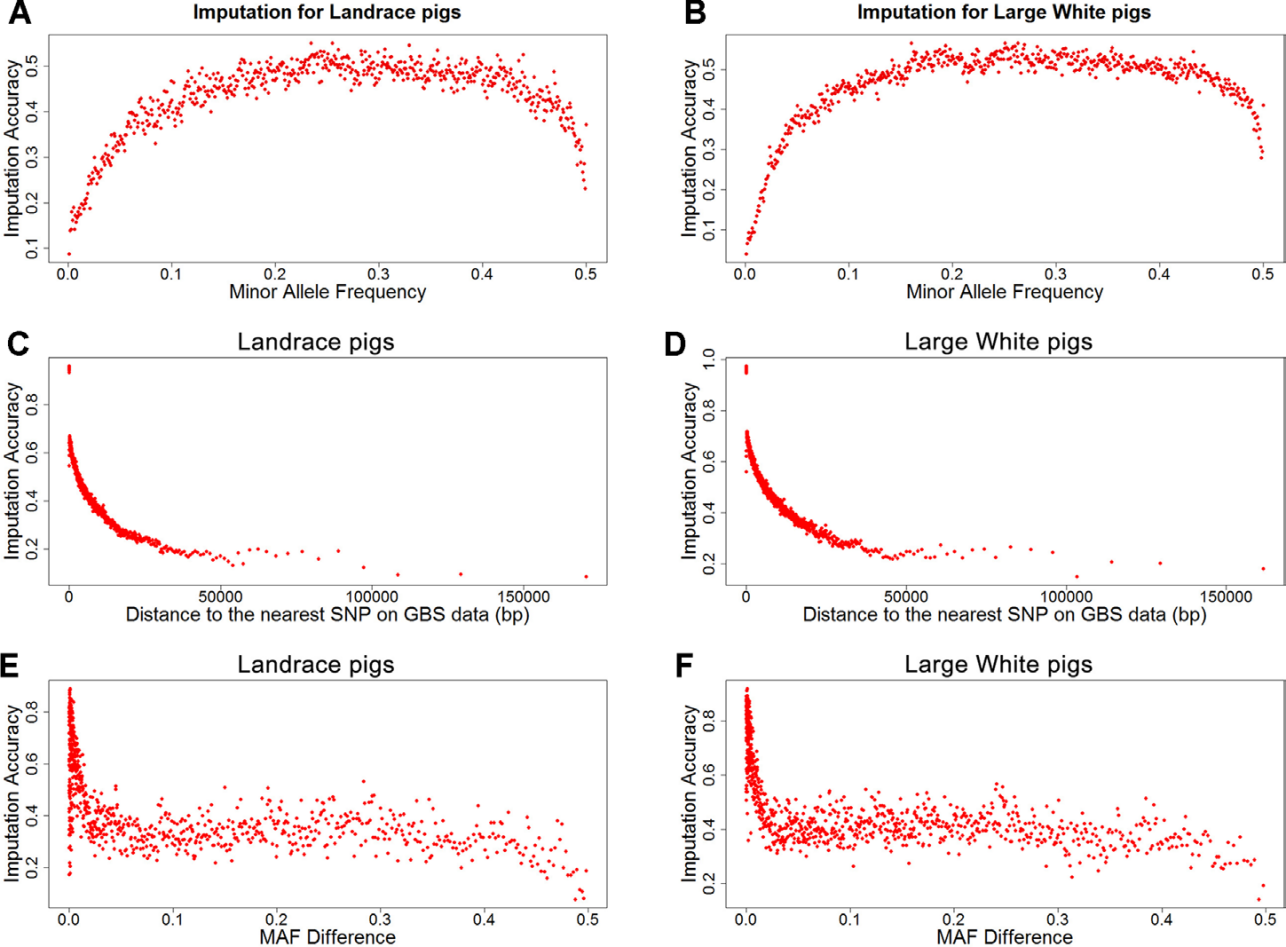
Average imputation accuracy versus minor allele frequency of imputed SNPs, distance, and MAF difference between the imputed SNPs and their closest SNPs on GBS data for Landrace **(A**, **C**, and **E)** and Large White pigs **(B**, **D** and **F)**. SNPs were grouped in bins of 1,000 SNPs with similar MAF differences.

To investigate factors that affect imputation accuracy, the distance and MAF difference between an imputed SNP and its closest SNP on the GBS data and MAF of imputed SNPs were analyzed. **Figures 2A, B** showed the average imputation accuracy versus MAF of imputed SNPs for the two populations. The average imputation accuracy was poor for SNPs with MAF smaller than 0.1 in two breeds. The average imputation accuracy was comparatively stable at MAF 0.10 to 0.45, but there was a temporary reduction when the MAF approached 0.5. After quality control, the average imputation accuracy with different MAFs was stable and both close to 0.72 (0.93 for accuracy > 0.8) in two breeds (**Supplementary Figure 1**). The large distance and MAF difference between imputed SNPs and their nearest SNPs on GBS data would result in a low imputation accuracy. The average imputation accuracy decreased with increasing distance and MAF difference, as illustrated by chromosome 1 for two breeds (**Figures 2C–F**).

### Whole-Genome Resequencing Association Analyses

This study performed GWAS on two target populations (Landrace and Large White pigs) in three scenarios, using data before and after imputation. In the first scenario, using the GBS data, GWAS was conducted for each breed. In the second scenario, using imputed WGS data (imputation accuracy > 0.3), GWAS was conducted for each breed. In the third scenario, imputed WGS data with accuracy higher than 0.8 were used in association analyses for each breed.

### GWAS for Data Before Imputation

**Figure 3:**
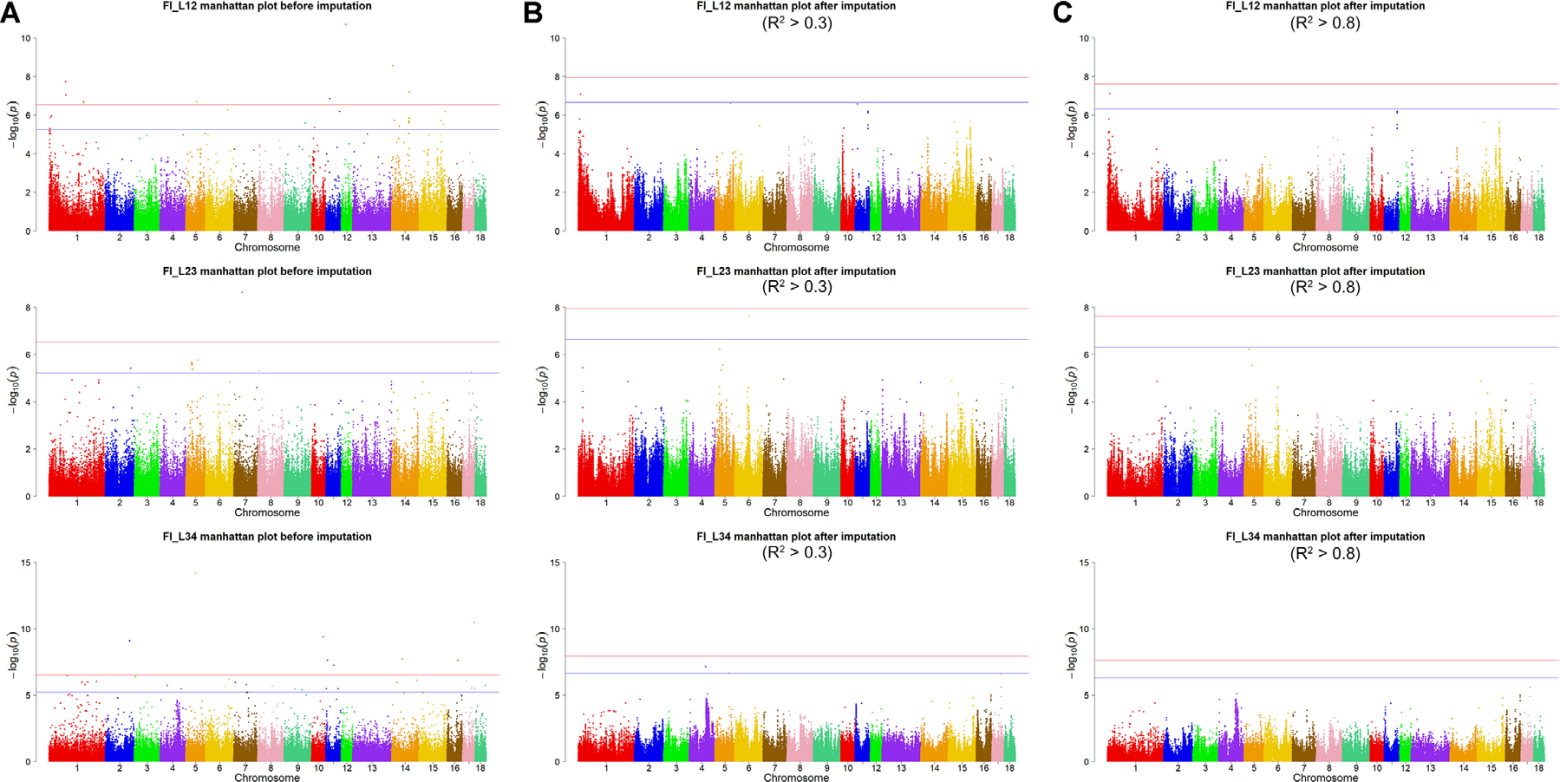
Manhattan plots of association results for FI of different parities using different SNP data **(A)** GBS data, **(B)** imputed WGS data with allelic *R* ^2^ >0.3, **(C)** imputed WGS data with allelic *R* ^2^ > 0.8) in Landrace pigs.

Using the original GBS data, this study conducted single-marker association studies for FI in each parity and each population. For Landrace pigs, the Manhattan plots are shown in **Figure 3A**. The Q-Q plots are shown in **Supplementary Figure 2A**, and the genomic inflation factors were between 0.99 and 1.05 (**Supplementary Table 5**). A total of 18 genome-wide significant SNPs were associated with FI, including nine SNPs for FI_L12, one for FI_L23, and eight for FI_L34 (**Table 2**). These significant SNPs were distributed on SSC1, SSC5, SSC11, SSC12, and SSC14. The most significant loci (the top SNP SSC12: 24,879,958 bp, *P* = 2.04 × 10^–11^) were located in the region of SSC12: 24.86 to 24.90 Mb, and four candidate genes were found in this region. Moreover, totals of 19, 17, and 27 suggestive SNPs were identified for FI_L12, FI_L23, and FI_L34 in Landrace pigs (**Supplementary Table 2**), respectively. At the suggestive level, three chromosome regions with five consecutive SNPs were found in this study. The first region with the top SNP SSC14: 97,176,453 bp (*P* = 2.35 × 10^–6^) was located in SSC14: 97.16 to 97.20 Mb for FI_L12. The second region was located in SSC5: 33.86 to 33.90 Mb for FI_L23; the top SNP at this location was SSC5: 33,882,769 bp (*P* = 2.09 × 10^–6^). The third region with the top SNP SSC5: 36,749,766 bp (*P* = 2.83 × 10^–6^) was located in SSC5: 36.73 to 36.77 Mb for FI_L23.

**Table 2:** The GWAS results at genome significant level for FI of different parities using GBS data in pigs.

Trait	Chr	Range of SNP (Mb)	Number of SNP	Top SNP position (bp)	n_miss	Allele	Allele frequency	Candidate gene	*P*
FI_L12	1	91.98–92.02	1	92,003,527	22	A/T	0.028		1.88E-08
FI_L12	1	94.76–94.80	1	94,779,648	22	A/T	0.012		9.18E-08
FI_L12	1	193.57–193.61	1	193,593,040	22	A/G	0.021		2.10E-07
FI_L12	5	60.57–60.61	1	60,589,650	23	T/G	0.045	ETV6	2.02E-07
FI_L12	11	22.40–22.44	1	22,424,991	19	G/A	0.042		1.45E-07
FI_L12	12	24.86–24.90	1	24,879,958	13	G/T	0.027	HOXB7/HOXB8/HOXB9/MIR196A-1	2.04E-11
FI_L12	14	4.88–4.92	1	4,904,707	10	C/A	0.011		2.73E-09
FI_L12	14	97.23–97.27	2	97,251,403	19	A/G	0.026		6.52E-08
FI_L23	7	46.65–46.69	1	46,665,496	6	G/A	0.017	GSTA4/RF00100/ICK	2.24E-09
FI_L34	2	135.91–135.95	1	135,932,694	5	C/A	0.022		8.09E-10
FI_L34	5	55.26–55.30	1	55,275,110	7	T/A	0.013		6.38E-15
FI_L34	10	65.05–65.09	1	65,071,338	2	C/A	0.017	FBH1	3.87E-10
FI_L34	11	9.67–9.71	1	9,688,680	8	T/C	0.022		2.35E-08
FI_L34	11	45.04–45.08	1	45,058,333	5	G/T	0.03	BORA/DIS3/PIBF1	5.34E-08
FI_L34	14	59.76–59.80	1	59,780,380	9	C/T	0.022		1.83E-08
FI_L34	16	60.14–60.18	1	60,161,199	9	C/T	0.022		2.30E-08
FI_L34	17	63.80–63.84	1	63,824,477	6	C/T	0.017		3.25E-11
FI_Y12	1	162.16–162.20	2	162,179,241	6	C/T	0.016	ALPK2	1.91E-08
FI_Y12	1	162.20–162.24	3	162,222,395	0	A/G	0.016	ALPK2	2.22E-08
FI_Y12	1	162.36–162.40	1	162,375,129	0	G/A	0.016	NEDD4L	2.22E-08
FI_Y12	1	162.39–162.43	2	162,406,916	18	G/A	0.015	RF00100	2.61E-08
FI_Y12	1	162.44–162.48	1	162,458,116	4	G/A	0.016		2.45E-08
FI_Y12	1	162.51–162.55	1	162,528,642	1	T/G	0.016		2.29E-08
FI_Y12	1	162.55–162.59	1	162,569,732	17	G/A	0.015		2.12E-08
FI_Y12	1	162.90–162.94	1	162,919,642	1	T/C	0.016	ATP8B1	2.23E-08
**FI_Y12**	**2**	**136.11–136.15**	**1**	**136,127,645**	**1**	**A/G**	**0.012**		**6.04E-11**
FI_Y12	3	106.83–106.87	3	106,849,597	10	G/A	0.011	TTC27	1.89E-11
FI_Y12	3	107.26–107.30	4	107,281,336	0	G/A	0.011	BIRC6	2.13E-11
FI_Y12	3	109.38–109.42	3	109,400,403	2	T/C	0.012		2.00E-11
FI_Y12	4	111.53–111.57	1	111,553,999	12	C/T	0.013		1.09E-09
FI_Y12	4	38.34–38.38	1	38,362,147	23	T/G	0.013	KCNS2	3.68E-08
**FI_Y12**	**5**	**103.41–103.45**	**1**	**103,426,443**	**9**	**A/C**	**0.013**		**5.22E-16**
FI_Y12	5	55.47–55.51	1	55,486,024	13	A/C	0.013	RF00026	1.11E-09
**FI_Y12**	**6**	**27.78–27.82**	**4 (including 27,811,226)**	**27,804,546**	**0**	**T/C**	**0.022**	**HSF4/B3GNT9/TRADD/NOL3/KIAA0895L/C16orf70**	**2.56E-08**
FI_Y12	7	2.04–2.08	9	2,061,622	0	A/G	0.012	SLC22A23	3.32E-10
FI_Y12	7	2.14–2.18	3	2,161,296	3	T/A	0.016		6.46E-11
FI_Y12	7	2.20–2.24	1	2,216,046	4	G/A	0.02		6.08E-08
FI_Y12	7	2.20–2.24	13	2,221,168	0	G/A	0.014	PXDC1	9.60E-09
FI_Y12	7	2.27–2.31	4	2,289,143	18	G/C	0.024	FAM50B	1.55E-07
FI_Y12	7	2.54–2.58	1	2,557,543	3	T/C	0.014		9.93E-09
FI_Y12	7	2.72–2.76	1	2,737,334	2	A/G	0.014		9.94E-09
FI_Y12	7	2.77–2.81	2	2,790,067	21	G/A	0.015		1.33E-08
FI_Y12	7	2.93–2.97	2	2,946,286	7	C/A	0.014	CDYL	1.12E-08
FI_Y12	7	2.96–3.00	2	2,984,884	6	G/A	0.014	PPP1R3G/RPP40	1.07E-08
FI_Y12	7	3.09–3.13	1	3,113,873	1	A/G	0.014		9.62E-09
FI_Y12	7	3.25–3.29	2	3,273,858	5	G/A	0.014	FARS2	9.82E-09
FI_Y12	7	3.34–3.38	1	3,358,876	2	G/T	0.014	FARS2	2.36E-08
FI_Y12	7	4.34–4.38	1	4,362,436	0	C/G	0.016		7.90E-08
FI_Y12	7	4.44–4.48	3	4,463,468	12	T/C	0.025		7.57E-08
FI_Y12	7	4.83–4.87	1	4,847,230	1	T/A	0.016	DSP	8.17E-08
FI_Y12	7	126.22–126.26	1	126,240,205	0	A/G	0.012		7.36E-10
FI_Y12	7	133.35–133.39	1	133,368,593	0	A/G	0.498		3.00E-20
FI_Y12	8	144.75–144.79	1	144,770,928	21	T/C	0.018		9.09E-08
**FI_Y12**	**10**	**3.59–3.63**	**2**	**3,609,429**	**24**	**C/T**	**0.031**		**3.40E-12**
FI_Y12	10	43.64–43.68	1	43,663,373	17	C/A	0.017	ST8SIA6	4.56E-08
FI_Y12	11	73.33–73.37	1	73,353,457	9	C/A	0.017		1.26E-07
FI_Y12	12	2.80–2.84	1	2,824,213	15	A/G	0.026	RBFOX3	4.59E-08
FI_Y12	13	209.06–209.10	3	209,081,546	1	A/C	0.011		5.01E-11
FI_Y12	14	15.45–15.49	2	15,472,751	1	A/G	0.018	GLRA3	1.14E-09
**FI_Y12**	**14**	**15.18–15.22**	**1**	**15,199,253**	**3**	**A/T**	**0.024**		**8.60E-09**
FI_Y12	15	4.07–4.11	2	4,094,589	0	A/G	0.026	ORC4	5.84E-09
FI_Y12	15	4.10–4.14	1	4,121,092	4	A/G	0.02		2.40E-07
FI_Y12	15	4.47–4.51	1	4,487,592	24	C/G	0.02		2.49E-08
FI_Y12	15	4.88–4.92	1	4,895,899	0	T/A	0.022		6.28E-08
FI_Y12	15	5.22–5.26	6	5,235,766	6	G/A	0.023		7.35E-08
FI_Y12	15	134.32–134.36	1	134,340,038	21	C/A	0.013		2.74E-10
**FI_Y12**	**15**	**150.28–150.32**	**1**	**150,297,519**	**6**	**T/C**	**0.019**		**4.32E-13**
FI_Y12	16	65.60–65.64	1	65,618,538	14	A/G	0.023	LSM11/THG1L	2.26E-07
FI_Y12	17	16.52–16.56	3	16,543,976	20	G/T	0.015		3.76E-09
FI_Y12	17	63.20–63.24	1	63,224,611	18	T/G	0.058		6.51E-08
FI_Y12	18	0.05–0.09	1	65,826	2	C/T	0.038		1.92E-07
FI_Y23	4	81.79–81.83	1	81,813,944	18	T/G	0.011	NME7	6.99E-10
FI_Y23	4	73.90–73.94	1	73,919,093	14	A/C	0.011	TOX	3.35E-08
FI_Y23	4	127.80–127.84	1	127,815,487	9	C/A	0.013		1.51E-07
FI_Y23	6	114.17–114.21	1	114,189,381	23	T/C	0.011		1.38E-07
FI_Y23	7	46.65–46.69	1	46,665,504	22	G/T	0.014	GSTA4/RF00100/ICK	8.67E-10
FI_Y23	9	46.50–46.54	1	46,524,685	16	G/T	0.018	CBL/MCAM/RNF26	8.49E-08
FI_Y23	11	65.25–65.29	1	65,267,206	8	C/A	0.011	DNAJC3	5.23E-09
FI_Y23	15	123.50–123.54	1	123,522,220	18	G/A	0.044	EPHA4	3.29E-09
FI_Y34	2	62.61–62.65	1	62,633,529	3	T/C	0.498	SLC1A6/LOC100736663/LOC100523890	1.00E-07
FI_Y34	7	133.40–133.44	5	133,416,643	1	T/C	0.498		9.88E-08
FI_Y34	9	33.26–33.30	1	33,275,594	3	T/C	0.041	MMP20	3.58E-08
FI_Y34	11	0.60–0.64	1	616,632	20	C/A	0.013	ZMYM5	1.89E-08
FI_Y34	15	35.81–35.85	1	35,828,705	1	G/A	0.498		9.89E-08

Chr, chromosome; range of SNP, range of significant chromosome region; number of SNP, number of SNP involved; n_miss, number of missing values of the SNP; alleles, alleles of top SNP.

The bolded text shown that the common SNPs detected in both GBS data and imputed WGS data.

**Figure 4:**
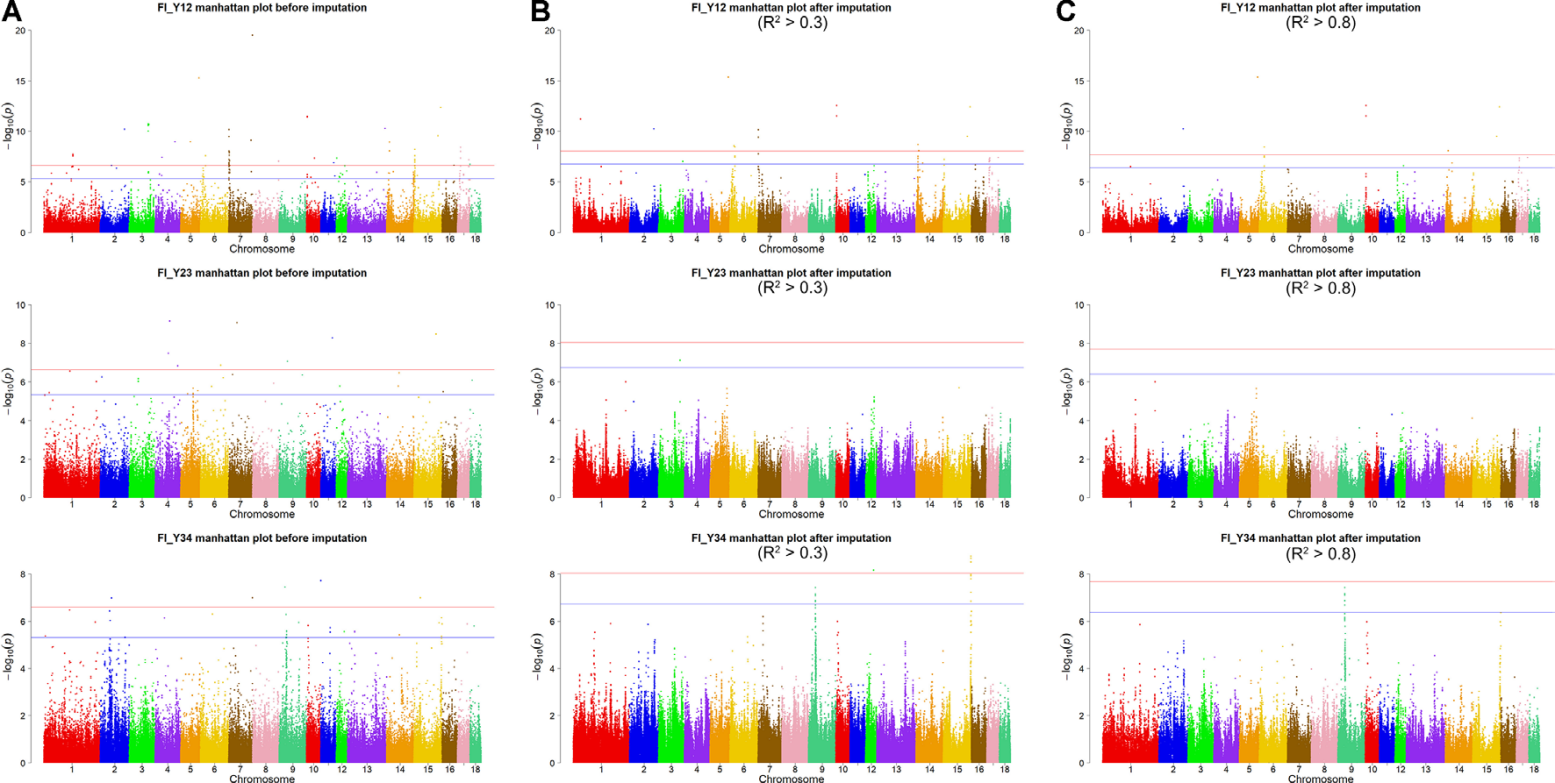
Manhattan plots of association results for FI of different parities using different SNP data **(A)** GBS data, **(B)** imputed WGS data with allelic *R* ^2^ > 0.3, **(C)** imputed WGS data with allelic *R* ^2^ > 0.8) in Large White pigs.

For Large White pigs, the Manhattan plot is shown in **Figure 4A**. The Q-Q plots are shown in **Supplementary Figure 3A**, and the genomic inflation factors were between 1.08 and 1.17 (**Supplementary Table 5**). Totals of 111, 8, and 9 genome-wide significant SNPs were associated with FI_Y12, FI_Y23, and FI_Y34 (**Table 2**), respectively. The significant chromosome regions on SSC7 (2.04–2.08, 2.20–2.24, and 133.40–133.44 Mb) and SSC15 (5.22–5.26 Mb) showed three clear signals. Nine consecutive SNPs were located in the region of SSC7: 2.04 to 2.08 Mb for FI_Y12, and the top SNP was SSC7: 2,061,622 bp (*P* = 3.32 × 10^–10^). In the region of SSC7: 2.20 to 2.24 Mb, the significant locus with the top SNP SSC7: 2,221,168 bp (*P* = 9.60 × 10^–9^) harbored 13 adjacent SNPs for FI_Y12. In addition, six significant SNPs were located in the region of 5.22 to 5.26 Mb on SSC15 for FI_Y12. For FI_Y34, five consecutive SNPs (the top SNP SSC7: 133,416,643 bp, *P* = 9.88 × 10^–8^) were detected in the region of 133.40 to 133.44 Mb on SSC7. At the suggestive level, totals of 99, 22, and 45 suggestive SNPs were detected for FI_Y12, FI_Y23, and FI_Y34 (**Supplementary Table 2**), respectively. Seven adjacent SNPs were located in the region of SSC7: 2.04 to 2.08 Mb, and the top SNP SSC7: 2,061,575 bp (*P* = 1.22 × 10^–6^) was associated with FI_Y12. In the region of SSC7: 2.23 to 2.27 Mb, five suggestive SNPs were found for FI_Y12. For FI_Y34, three chromosome regions with many consecutive SNPs were found, including the regions of SSC9: 38.19 to 38.23 Mb, SSC9: 41.07 to 41.11 Mb, and SSC13: 39.27 to 39.31 Mb. In the first region SSC9: 38.19 to 38.23 Mb, five SNPs were strongly associated with FI_Y34, and the top SNP was SSC9: 38,206,875 bp (*P* = 5.06 × 10^–7^). In the second region SSC9: 41.07 to 41.11 Mb, nine consecutive SNPs were detected, and the *P* value of the top SNP SSC9: 41,085,054 bp was 2.52 × 10^–6^. The locus with the top SNP, SSC13: 39,293,665 bp (*P* = 2.63 × 10^–6^), contained seven SNPs in total and was found to be associated with FI_Y34.

### GWAS for Data After Imputation

For imputed WGS with accuracy >0.3, in Landrace pigs, totals of 4,404,137, 4,402,124, and 4,380,454 SNPs were analyzed for FI_L12, FI_L23, and FI_L34, respectively. In Large White pigs, the totals were 5,486,741, 5,486,265, and 5,474,718 SNPs for FI_Y12, FI_Y23, and FI_Y34, respectively (**Supplementary Table 1**). Based on the genome-wide-significance threshold (0.05/*N*) and suggestive threshold (1/*N*), a total of 125 genome-wide significant SNPs and 259 suggestive SNPs were detected in two populations.

For Landrace pigs, the results are shown in **Figure 3B** and **Table 3**. The Q-Q plots are shown in **Supplementary Figure 2B**, and the genomic inflation factors were between 0.95 and 1.03 (**Supplementary Table 5**). No genome-wide significant loci were found for FI of each parity using imputed data. Meanwhile, a total of 15 suggestive loci (**Supplementary Table 3**) were detected for FI with *P* values between 2.13 × 10^–7^ and 2.41 × 10^–8^. Of these, one SNP was found for FI_L12, six SNPs for FI_L23, and eight SNPs for FI_L34. In the region SSC6: 77.40 to 77.44 Mb, six consecutive SNPs were associated with FI_L23, and the position of the top SNP was SSC6: 77,416,585 bp (*P* = 2.41 × 10^–8^). At 91.47 to 91.51 Mb on SSC4, seven SNPs associated with FI_L34 were found; the top one was SSC4: 91,492,978 bp (*P* = 6.98 × 10^–8^).

**Table 3:** The GWAS results at genome significant level for FI of different parities using imputed WGS data (allelic *R* ^2^ > 0.3) in pigs.

Trait	Chr	Range of SNP (Mb)	Number of SNP	Top SNP position (bp)	Allelic *R* ^2^	n_miss	Alleles	Allele frequency	Candidate gene	*P*
FI_Y12	1	39.39–39.43	2	39,407,333	0.65	0	A/G	0.014		6.08E-12
**FI_Y12**	**2**	**136.11–136.15**	**19**	**136,127,645**	**1.00**	**0**	**A/G**	**0.012**		**5.81E**-**11**
**FI_Y12**	**5**	**103.41–103.45**	**3**	**103,426,443**	**0.96**	**0**	**A/C**	**0.012**		**4.29E**-**16**
FI_Y12	6	23.29–23.33	6	23,313,531	0.75	0	T/C	0.044	CDH8	2.88E-09
**FI_Y12**	**6**	**27.79–27.83**	**1**	**27,811,226**	**0.93**	**0**	**A/G**	**0.02**	**C16orf70/B3GNT9/TRADD/HSF4/NOL3/KIAA0895L/EXOC3L1**	**3.68E**-**09**
FI_Y12	7	2.14–2.18	35	2,160,719	0.61	0	T/C	0.018		6.93E-11
**FI_Y12**	**10**	**3.59–3.63**	**4**	**3,609,429**	**0.88**	**0**	**T/C**	**0.026**		**2.81E**-**13**
FI_Y12	14	10.67–10.71	22	10,694,678	0.74	0	C/T	0.068	ADRA1A	2.00E-09
**FI_Y12**	**14**	**15.18–15.22**	**6**	**15,199,253**	**0.99**	**0**	**A/G**	**0.024**		**8.07E**-**09**
**FI_Y12**	**15**	**150.28–150.32**	**3**	**150,297,519**	**0.98**	**0**	**T/C**	**0.018**		**3.82E**-**13**
FI_Y12	15	134.32–134.36	1	134,340,038	0.90	0	C/A	0.012		3.18E-10
FI_Y34	12	43.71–43.75	11	43,730,214	0.69	0	G/A	0.014	NF1	6.79E-09
FI_Y34	15	154.76–154.80	1	154,777,447	0.61	0	T/G	0.159		2.17E-09
FI_Y34	15	154.77–154.81	1	154,788,901	0.62	0	T/C	0.159		2.17E-09
FI_Y34	15	154.91–154.95	10	154,933,940	0.75	0	C/T	0.212		1.72E-09

Chr, chromosome; range of SNP, range of significant chromosome region; number of SNP, number of SNP involved; allelic R2, estimated correlation between the imputed and true genotypes; n_miss, number of missing values of the SNP; alleles, alleles of top SNP.

The bolded text shown that the common SNPs detected in both GBS data and imputed WGS data.

For Large White pigs, the results are shown in **Figure 4B** and **Table 3**. The Q-Q plots are shown in **Supplementary Figure 3B**, and the genomic inflation factors were between 1.01 and 1.03 (**Supplementary Table 5**). A total of 125 genome-wide significant SNPs were identified, including 102 significant SNPs for FI_Y12 and 23 SNPs for FI_Y34 (**Table 3**). In addition, a total of 244 suggestive SNPs were found, including 111 SNPs for FI_Y12, 1 for FI_Y23, and 132 for FI_Y34 (**Supplementary Table 3**). Notably, four interesting peaks were observed, as shown in **Figure 4B**. A significant region at 2.14 to 2.18 Mb on SSC7 with the top SNP of SSC7: 2,159,059 bp (*P* = 6.93 × 10^–11^) was found for FI_Y12. At 10.67 to 10.71 Mb in SSC14, this study detected a significant locus (top SNP SSC14: 10,694,678 bp, *P* = 2.00 × 10^–9^) for FI_Y12. The third region was located in SSC15: 154.91 to 154.95 Mb with the top SNP SSC15: 154,933,940 bp (*P* = 1.72 × 10^–9^) for FI_Y34. The fourth region was located in SSC9: 38.20 to 38.24 Mb (**Supplementary Table 3**). In this region, a total of 119 consecutive SNPs and two candidate genes (*ZC3H12C* and *RDX* gene) for FI_Y34 were detected; the top SNP was located at SSC9: 38,215,712 bp (*P* = 3.74 × 10^–8^).

After filtering the imputed data to those with imputation accuracy of higher than 0.8, in Landrace pigs, totals of 2,043,321, 2,044,003, and 2,032,985 SNPs were analyzed for FI_L12, FI_L23, and FI_L34, respectively. In Large White pigs, the totals were 2,453,952, 2,453,789, and 2,449,239 SNPs for FI_Y12, FI_Y23, and FI_Y34, respectively (**Supplementary Table 1**). The GWAS results are shown in **Figure 3C**, **Figure 4C**, and **Table 4** and **Supplementary Table 4**. The Q-Q plots are shown in **Supplementary Figures 2C and 3C**, and the genomic inflation factors were between 0.94 and 1.03 (**Supplementary Table 5**). Based on the genome-wide significance threshold (0.05/*N*) and suggestive threshold (1/*N*), a total of 27 genome-wide significant SNPs for FI_Y12 (**Table 4**) were detected. In terms of suggestive SNPs, 1 SNP for FI_L12 (**Supplementary Table 4**) and 74 for FI_Y34 (**Supplementary Table 4**) were detected. For FI_Y34, a peak was observed in the region of 38.20 to 38.24 Mb, and the top SNP was located at SSC9: 38215712.

**Table 4:** The GWAS results at genome significant level for FI of different parities using imputed WGS data (allelic *R* ^2^ > 0.8) in pigs.

Trait	Chr	Range of SNP (Mb)	Number of SNP	Top SNP position (bp)	Allelic *R* ^2^	n_miss	Alleles	Allele frequency	Candidate gene	*P*
FI_Y12	2	136.11–136.15	10	136,127,645	1.00	0	A/G	0.012		1.54E-09
FI_Y12	5	103.41–103.45	3	103,426,443	0.96	0	A/C	0.012		5.44E-13
FI_Y12	6	27.79–27.83	1	27,811,226	0.93	0	A/G	0.02	C16orf70/B3GNT9/TRADD/HSF4/NOL3/KIAA0895L/EXOC3L1	3.44E-08
FI_Y12	10	3.59–3.63	4	3,609,429	0.88	0	T/C	0.026		3.66E-11
FI_Y12	14	15.18–15.22	6	15,199,253	0.99	0	A/G	0.024		6.32E-08
FI_Y12	15	150.28–150.32	3	150,297,519	0.98	0	T/C	0.018		3.82E-13

Chr, chromosome; range of SNP, range of significant chromosome region; number of SNP, number of SNP involved; allelic R2, estimated correlation between the imputed and true genotypes; n_miss, number of missing values of the SNP; alleles, alleles of top SNP.

### Comparing GWAS Results for GBS Data and Imputed Data

In this study, a total of six genome-wide significant loci were simultaneously uncovered for genotype data both before and after imputation. In the region of 136.11 to 136.15 Mb, the first locus with the top SNP SSC2: 136,127,645 bp was detected, and the *P* values of the top SNP were 6.04 × 10^–11^ and 5.81 × 10^–11^ for GBS data and imputed data, respectively. The second locus was located in the region 103.41 to 103.45 Mb on SSC5. The *P* values of the top SNP SSC5: 103,426,443 bp in the data before and after imputation were 5.22 × 10^–16^ and 4.29 × 10^–16^, respectively. The third locus was located in the region of SSC15: 27.79 to 27.83 Mb; the position of the top SNP was SSC6: 27,811,226 bp (*P* = 2.56 × 10^–8^ for original data; *P* = 3.44 × 10^–8^ for imputed data). In the region of SSC10: 3.59 to 3.63 Mb, the fourth common top SNP SSC10: 3,609,429 bp was found, with *P* values of 3.40 × 10^–12^ (original) and 2.81 × 10^–13^ (imputed). The fifth locus was found in the region of SSC14: 15.18 to 15.22 Mb, and the *P* values of the top SNP SSC14: 15,199,253 bp were 8.60 × 10^–9^ (original) and 8.07 × 10^–9^ (imputed). At the sixth locus, the *P* values of the top SNP SSC15: 150,297,519 bp were 4.32 × 10^–13^ and *P* = 3.82 × 10^–13^ for the data before and after imputation, respectively. Moreover, a clear peak (SSC9: 38.20–38.24 Mb) was observed in the imputed data with accuracy higher than 0.3 and 0.8.

### Candidate Genes

The GWAS results using imputed data were used to detect functional genes in this study. Based on the pig genes Sscrofa11.1, a total of 10 candidate genes were detected near (within 20 Kb) the genome significant SNPs for imputed data. And, within a 20-Kb region centering each suggestive SNP, 20 genes were found for imputed data. These genes were used to conduct GO analyses in DAVID software. A total of 210 GO terms were detected, of which four significant GO terms (*P* < 0.05) were identified. The significant GO terms were associated with cellular response to heat, secretory granule, regulation of gene expression, and response to hypoxia. Considering the genes involved in these significant GO terms and functional annotation in NCBI database and reported literatures, *CHST11, NF1*, and *ADRA1A* promising candidate genes were suggested for FI.

## Discussion

This is the first study to investigate the imputation accuracy from GBS to WGS data and perform GWAS using the GBS and imputed WGS data for FI of different parities in Landrace and Large White pigs. The previous study demonstrated that using multiple reference populations, the imputation from low-density SNPs chip to WGS resulted in a poor imputation accuracy in pigs (Berg et al., 2019). Thus, 20 Landrace and 40 Large White pigs were separately used as a single-breed reference population for each breed. A total of 300 Landrace and 300 Large White pigs were separately used as the target population in this study. Subsequently, the imputations were conducted for each breed by Beagle software. In summary, GWAS using the imputed WGS demonstrated that use of imputed WGS would improve identification of genetic variants. The imputation accuracy and GWAS results were discussed in the following sections.

### Imputation From GBS to WGS

Imputation accuracy is known to be affected by the size of the reference population, population structure, imputation method, and marker density (Ye et al., 2018). To investigate the accuracy of imputation to WGS in Landrace and Large White pigs, the GBS data were directly imputed to WGS data, using a single-breed reference population with Beagle software. After imputation, 35.17% to 42.10% of SNPs had imputation accuracy higher than 0.3. Differences in the reference population size would result in differences in imputation accuracy. Specifically, a limited reference population would result in poor imputation accuracy (Binsbergen et al., 2014). In our study, the average imputation accuracy was higher for Large White pigs than for Landrace pigs. In addition, for most chromosomes (with the exceptions of chromosomes 3, 10, and 16), the accuracy for each chromosome was also higher for Large White pigs than for Landrace pigs. This can be explained by there being only 20 Landrace pigs in the reference population compared with 40 Large White pigs. These results demonstrate that the reference population size contributed to imputation accuracy and that imputation accuracy increased with increasing population size. In addition, the differences in population structure and genetic architecture between Large White and Landrace pigs probably also resulted in the differences in imputation accuracy between two breeds. These factors would result in different rates of LD decay and different numbers of independent chromosome segments (Goddard et al., 2009). Lower LD decay and a high rate of shared haplotypes would result in high imputation accuracy.

Using Beagle software, it was reported that the rates of imputation accuracy from BovineHD bead chip to WGS were 0.77 to 0.83 in bovines (Binsbergen et al., 2014). In addition, in a study by Ni et al. (2015) on chicken, the imputation accuracy from a 600 K chip to WGS data was found to be more than 0.95. The imputation accuracy was also reported to increase with increasing density of the target SNP chip, sequencing cost, number of reference individuals (Ye et al., 2018), and MAF (Berg et al., 2019). In that study, it was found that accuracy of imputation from 60- and 600-K chip data to WGS data was 0.62 and 0.81, while the accuracy ranged from 0.421 to 0.897 for 1 to 24 reference individuals in hens (Ye et al., 2018). However, imputation accuracy in this study was similar to that in a study with 90 reference individuals (accuracy of 0.46) in bulls (Binsbergen et al., 2014). Expanding the reference population size would improve imputation accuracy (Bouwman and Veerkamp, 2014). In comparison to these previous studies, the imputation accuracy from GBS to WGS data was relatively low in our study. Possible reasons for this include the limited reference population (reference populations of 20 and 40 individuals) and the low-density target genotypes (about 320-K genotypes).

Quantitative traits are controlled by few genes with large effects and numerous polygenes with minor effects. Loci with a low allele frequency may have large effects on the complex traits (Manolio et al., 2009). However, average imputation accuracy was quite low for the genetic variants with lower MAF. Indeed, accuracy is generally lower for SNPs with low MAF. Factors that could affect imputation accuracy are MAF of imputed SNPs, distance, and MAF difference between the imputed SNPs and their nearest SNPs on target data. As expected, the average imputation accuracy sharply decreased for SNPs with MAF of imputed SNPs below 0.1, while it was comparatively stable at MAF of imputed SNPs 0.1 to 0.45 in this study. These findings are in agreement with the literatures (Chen and He, 2012; Hayes et al., 2012; Ye et al., 2018; Bolormaa et al., 2019). In another study, the imputation accuracy showed a sharp decline when MAF was smaller than 0.2 in hens (Ye et al., 2018). However, the average imputation accuracy was here found to decrease at MAF > 0.45. The large distance and MAF difference would result in a low imputation accuracy (Binsbergen et al., 2014; Berg et al., 2019). In this study, the distance and MAF difference between imputed SNPs and their closest SNPs on GBS data were large at MAF <0.1 and MAF > 0.45 in two populations (**Supplementary Figures 4 and 5**). And the average imputation accuracy decreased with increasing distance and MAF difference. Thus, the low average imputation accuracy at MAF < 0.1 and MAF > 0.45 may have been caused by the large distance and MAF difference between imputed SNPs and their closest SNPs on GBS data.

Furthermore, the appropriate selection of key individuals used as a reference population would contribute to increasing the accuracy for low-MAF SNPs compared with random selection (Pausch et al., 2013; Moghaddar et al., 2015; Ye et al., 2018). However, the use of a reference population with the closest relationship would result in a lower imputation accuracy than random selection (Yu et al., 2014). In this study, random selection of the reference population resulted in poor imputation accuracy. In further study, the appropriate selection of key individuals for sequencing should be performed to determine whether it can contribute to increasing the accuracy of imputation from low-density data to WGS data in pigs.

### The Identified QTLs and Potential Candidate Genes

Based on low-density SNP chips, association analyses achieved low power and accuracy for detecting loci associated with complex traits. Using WGS data, GWAS is optimized to identify genetic variants for complex traits. However, the imputation from GBS to WGS data obtained a poor imputation accuracy. The imputed WGS data contained the most causal loci. The use of imputed WGS data instead of GBS data improved the identification of loci of interest. In this study, association analyses using imputed WGS data rather than GBS data could reduce the mapping noise and highlight the peaks that are important for FI. All of the SNPs for imputed WGS with accuracy higher than 0.8 matched those in the imputed WGS with accuracy higher than 0.3.

In this imputed GWAS, a total of 90 significant SNPs associated with FI_Y12 were located on 12 QTL regions reported to be associated with reproductive traits, including age at puberty (Nonneman et al., 2014), gestation length (Wilkie et al., 1999; Chen et al., 2010), corpus luteum number (Rohrer et al., 1999; Schneider et al., 2014), teat number (Hernandez et al., 2014), litter size (Hernandez et al., 2014), number of stillborn (Onteru et al., 2012), and number of offspring born alive (Tribout et al., 2008). For FI_Y34, the peak SNP (SSC12: 43730214) was included within seven reproduction-related QTL regions associated with teat number (Hirooka et al., 2001) and gestation length (Chen et al., 2010), in which the NF1 gene was found. This study further confirmed the importance of these 19 QTLs in pigs.

GWAS with imputed WGS data may be effective to detect putative candidate genes. Numerous putative candidate genes for FI located near these identified loci were found in two populations. Of these, the product of the *CHST11* gene is localized at the Golgi membrane and is a key enzyme in the biosynthesis of chondroitin sulfates (Mikami et al., 2003). This gene was previously found to be highly expressed in ovarian cancer (Oliveira-Ferrer et al., 2015). The GO terms of the *CHST11* gene are related to chondrocyte development, postembryonic development, embryonic digit morphogenesis, developmental growth, and embryonic viscerocranium morphogenesis. The *NF1* gene is known as a key transcription factor that modulates the tissue-specific transcription of various genes (Ivanov et al., 1990). In bovines, the expression of the *NF1* gene was also found to dramatically increase in the development of lactation (Ivanov et al., 1990). The *NF1* gene also plays a significant role during embryogenesis in mice (Gutmann et al., 1995). Moreover, studies involving knockout of the *NF1* gene suggested that this gene significantly affects osteoblast development in embryonic stem cells (Yu et al., 2010) and is expressed in lactation and mammary glands. The *ADRA1A* gene is a member of the G protein–coupled receptor superfamily and modulates the mitogenic response and growth and proliferation of cells. It encodes the α-epinephrine receptor for epinephrine, norepinephrine, and catecholamine (Freitas et al., 2008) and plays an important role in smooth muscle contraction, myocardial inotropism, and hepatic glucose metabolism (Stéphane et al., 2007). Furthermore, this gene was found to play an important role in fetal sheep (Giussani et al., 1995). Therefore, investigation into the molecular mechanisms associated with these identified loci and genes could provide valuable insight into the genetic architecture behind FI in pigs and help to improve pig breeding.

## Conclusion

Using a single-breed reference population, imputation from GBS to WGS data resulted in a poor imputation accuracy. After imputation, the average imputation accuracy (allelic *R* ^2^) was 0.42 and 0.45 for Landrace and Large White pigs, respectively. The different size of reference population may have contributed to this difference of imputation accuracy in Landrace and Large White pigs. Although the imputation accuracy was low, the imputed WGS data promoted the detection of loci affecting quantitative traits. The use of the imputed WGS for GWAS appeared to reduce the mapping noise and highlight the important peaks in this study. These results provide useful novel insight into the genetic variants and genes associated with FI of different parities in Landrace and Large White pigs. However, further studies are needed to determine the optimal imputation strategy from GBS to WGS data and to validate these identified SNPs and genes.

## Data Availability Statement

The datasets supporting the results of this article are included within the article. All genotypic and phenotype data were deposited in Figshare DOI: 10.6084/m9.figshare.9505259.

## Ethics Statement

All experimental procedures were performed in accordance with the Institutional Review Board (IRB14044) and the Institutional Animal Care and Use Committee of the Sichuan Agricultural University under permit number DKY-B20140302.

## Author Contributions

PW, GT, and KW performed experiments. PW, KW and JZ analyzed data and prepared figures and tables. GT and PW edited and revised manuscript. GT, PW, YL, BF, AJ, LS, WX, YJ, LZ, YZ, XX and XL conceived, designed research and wrote this paper. PW, KW, JZ, DC, QY, XY, YL, BF, AJ, LS, WX, YJ, LZ, YZ, XX, XL and GT approved final version of this manuscript.

## Funding

The study was supported by grants from the National key R&D Program of China #2018YFD0501204, the National Natural Science Foundation of China (31530073), the National Natural Science Foundation of China #C170102, the Chinese National Science and Tech Support Program (no. 2015BAD03B01, 2015GA810001), and the earmarked fund for the China Agriculture Research System (no. CARS-35-01A).

## Conflict of Interest

The authors declare that the research was conducted in the absence of any commercial or financial relationships that could be construed as a potential conflict of interest.
