# Supplementary material for: GWAS on Imputed Whole-Genome Resequencing From Genotyping-by-Sequencing Data for Farrowing Interval of Different Parities in Pigs
Source: Front Genet. 2019 Oct 18;10:1012. doi: 10.3389/fgene.2019.01012 (PMC6813215; doi:10.3389/fgene.2019.01012)
Supplement: Supplementary file 1 [file DataSheet_1.docx]

**Additional files**

Supplementary Table 1. The Number of analyzed SNPs for GWAS in FI traits of different parities.

| Before imputation | | After imputation (R^2^ > 0.3) | | After imputation (R^2^ > 0.8) | |
| --- | --- | --- | --- | --- | --- |
| Traits | Number of analyzed SNPs (N) | Traits | Number of analyzed SNPs (N) | Traits | Number of analyzed SNPs (N) |
| FI_L12 | 172,249 | FI_L12 | 4,404,137 | FI_L12 | 2,043,321 |
| FI_L23 | 167,047 | FI_L23 | 4,402,124 | FI_L23 | 2,044,003 |
| FI_L34 | 166,737 | FI_L34 | 4,380,454 | FI_L34 | 2,032,985 |
| FI_Y12 | 206,482 | FI_Y12 | 5,486,741 | FI_Y12 | 2,453,952 |
| FI_Y23 | 207,349 | FI_Y23 | 5,486,265 | FI_Y23 | 2,453,789 |
| FI_Y34 | 202,845 | FI_Y34 | 5,474,718 | FI_Y34 | 2,449,239 |

Supplementary Table 2. The suggestive SNPs using GBS data for FI trait of different parities in Landrace and Large White pigs.

| Traits | Chromosome | Range of SNP (Mb) | Number of SNP | Top SNP Position (bp) | n_miss | Allele | Allele Frequency | Candidate gene | P-value |
| --- | --- | --- | --- | --- | --- | --- | --- | --- | --- |
| FI-L12 | 1 | 13.61-13.65 | 1 | 13627542 | 1 | C/G | 0.011 | VIP | 1.12E-06 |
| FI-L12 | 1 | 7.35-7.39 | 1 | 7370803 | 6 | G/A | 0.024 | SLC22A1/IGF2R | 1.30E-06 |
| FI-L12 | 1 | 4.00-4.04 | 1 | 4023790 | 1 | A/G | 0.039 |  | 5.08E-06 |
| FI-L12 | 6 | 124.28-124.32 | 1 | 124302059 | 21 | T/C | 0.014 |  | 5.69E-07 |
| FI-L12 | 9 | 117.06-117.10 | 1 | 117083602 | 21 | T/A | 0.023 | CACYBP/MRPS14 | 2.61E-06 |
| FI-L12 | 10 | 16.41-16.45 | 1 | 16427079 | 3 | G/A | 0.013 | SDCCAG8/AKT3 | 4.45E-06 |
| FI-L12 | 11 | 78.60-78.64 | 1 | 78623212 | 8 | T/G | 0.02 | LAMP1/GRTP1 | 6.72E-07 |
| FI-L12 | 14 | 97.25-97.29 | 1 | 97274472 | 14 | G/A | 0.032 |  | 1.46E-06 |
| FI-L12 | 14 | 15.49-15.53 | 1 | 15511680 | 5 | A/T | 0.013 | GLRA3 | 1.88E-06 |
| FI-L12 | 14 | 96.34-96.38 | 1 | 96359595 | 1 | A/G | 0.028 | PCDH15 | 2.00E-06 |
| FI-L12 | 14 | 97.16-97.20 | 5 | 97176453 | 9 | T/C | 0.029 |  | 2.35E-06 |
| FI-L12 | 14 | 42.69-42.73 | 1 | 42707135 | 5 | A/C | 0.017 |  | 3.84E-06 |
| FI-L12 | 15 | 146.14-146.18 | 1 | 146156282 | 8 | T/G | 0.029 |  | 6.56E-07 |
| FI-L12 | 15 | 122.81-122.85 | 1 | 122828857 | 16 | C/G | 0.17 |  | 2.04E-06 |
| FI-L12 | 15 | 136.87-136.91 | 1 | 136888592 | 23 | A/G | 0.024 |  | 3.04E-06 |
| FI-L23 | 2 | 142.54-142.58 | 1 | 142557580 | 16 | T/A | 0.015 | PCDHAC2/PCDHA11/PCDHA13 | 3.80E-06 |
| FI-L23 | 5 | 69.10-69.14 | 1 | 69118218 | 4 | T/A | 0.02 |  | 1.71E-06 |
| FI-L23 | 5 | 33.86-33.90 | 5 | 33882769 | 2 | G/C | 0.011 | CCT2/LRRC10 | 2.09E-06 |
| FI-L23 | 5 | 36.00-36.04 | 1 | 36024425 | 0 | C/T | 0.011 | TPH2 | 2.38E-06 |
| FI-L23 | 5 | 33.84-33.88 | 1 | 33861344 | 14 | G/A | 0.012 | FRS2/RF00402 | 2.56E-06 |
| FI-L23 | 5 | 36.73-36.77 | 5 | 36749766 | 8 | C/A | 0.012 |  | 2.83E-06 |
| FI-L23 | 5 | 37.49-37.53 | 1 | 37513138 | 10 | T/C | 0.012 |  | 4.24E-06 |
| FI-L23 | 8 | 6.25-6.29 | 1 | 6267908 | 0 | T/C | 0.101 | SLC2A9 | 4.96E-06 |
| FI-L23 | 17 | 48.51-48.55 | 1 | 48528831 | 5 | T/A | 0.04 | TP53RK/SLC35C2/ELMO2 | 5.52E-06 |
| FI-L34 | 1 | 101.23-101.27 | 1 | 101250068 | 7 | C/A | 0.026 |  | 3.42E-07 |
| FI-L34 | 1 | 266.40-266.44 | 4 | 266415272 | 11 | T/C | 0.014 | PBX3 | 9.07E-07 |
| FI-L34 | 1 | 216.29-216.33 | 1 | 216311658 | 5 | G/T | 0.026 | ERMP1 | 1.02E-06 |
| FI-L34 | 1 | 183.05-183.09 | 1 | 183069754 | 6 | A/C | 0.013 |  | 1.05E-06 |
| FI-L34 | 1 | 201.55-201.59 | 1 | 201568760 | 7 | C/T | 0.013 |  | 1.56E-06 |
| FI-L34 | 3 | 5.57-5.61 | 1 | 5590490 | 4 | C/A | 0.013 |  | 3.92E-07 |
| FI-L34 | 4 | 39.57-39.61 | 1 | 39589745 | 3 | G/T | 0.013 | CPQ | 1.87E-06 |
| FI-L34 | 4 | 118.41-118.45 | 1 | 118427684 | 7 | C/T | 0.018 | PALMD | 3.42E-06 |
| FI-L34 | 6 | 130.11-130.15 | 1 | 130132775 | 9 | T/G | 0.013 |  | 6.26E-07 |
| FI-L34 | 6 | 112.09-112.13 | 1 | 112109811 | 3 | T/G | 0.013 |  | 2.13E-06 |
| FI-L34 | 7 | 8.82-8.86 | 1 | 8835670 | 10 | G/T | 0.014 |  | 1.10E-06 |
| FI-L34 | 7 | 69.33-69.37 | 1 | 69350329 | 7 | C/A | 0.026 |  | 1.59E-06 |
| FI-L34 | 8 | 81.84-81.88 | 1 | 81864617 | 7 | A/C | 0.013 | TTC29 | 2.03E-06 |
| FI-L34 | 9 | 60.35-60.39 | 1 | 60365059 | 12 | T/A | 0.028 |  | 3.54E-06 |
| FI-L34 | 9 | 99.97-100.01 | 1 | 99987878 | 8 | C/A | 0.013 |  | 4.09E-06 |
| FI-L34 | 11 | 68.09-68.13 | 1 | 68111572 | 10 | C/A | 0.023 |  | 3.17E-06 |
| FI-L34 | 11 | 3.91-3.95 | 1 | 3929927 | 8 | G/A | 0.031 | LOC100625134 | 3.21E-06 |
| FI-L34 | 14 | 140.98-141.02 | 1 | 140999328 | 11 | G/T | 0.014 | CFAP46 | 8.13E-07 |
| FI-L34 | 14 | 29.27-29.31 | 1 | 29292828 | 5 | G/T | 0.013 | ATP6V0A2/SUMO4/TCTN2 | 1.09E-06 |
| FI-L34 | 17 | 20.83-20.87 | 1 | 20853631 | 7 | G/T | 0.013 | BTBD3 | 9.23E-07 |
| FI-L34 | 17 | 50.02-50.06 | 2 | 50041147 | 10 | C/T | 0.041 |  | 2.96E-06 |
| FI-L34 | 17 | 69.08-69.12 | 1 | 69096725 | 5 | G/A | 0.022 |  | 3.15E-06 |
| FI-L34 | 18 | 58.22-58.26 | 1 | 58243037 | 6 | G/T | 0.03 |  | 1.88E-06 |
| FI-Y12 | 1 | 162.32-162.36 | 1 | 162340738 | 3 | T/C | 0.018 | ALPK2/ssc-mir-122 | 2.98E-07 |
| FI-Y12 | 1 | 155.40-155.44 | 1 | 155417340 | 24 | G/T | 0.013 |  | 3.38E-07 |
| FI-Y12 | 1 | 194.51-194.55 | 1 | 194529151 | 13 | C/T | 0.021 | PPP2R5E | 6.31E-07 |
| FI-Y12 | 1 | 126.59-126.63 | 2 | 126605808 | 1 | G/A | 0.02 | DUOX1/DUOXA2/DUOXA1/DUOX2 | 1.35E-06 |
| FI-Y12 | 1 | 41.24-41.28 | 1 | 41257848 | 20 | G/T | 0.02 | TBC1D32 | 1.40E-06 |
| FI-Y12 | 2 | 62.46-62.50 | 1 | 62482463 | 10 | G/C | 0.023 | OR1I1 | 2.48E-07 |
| FI-Y12 | 2 | 90.43-90.47 | 1 | 90452899 | 16 | T/G | 0.019 | ATG10 | 4.58E-07 |
| FI-Y12 | 3 | 108.17-108.21 | 2 | 108191912 | 1 | A/G | 0.077 | CAPN14 | 1.05E-06 |
| FI-Y12 | 3 | 106.31-106.35 | 1 | 106331155 | 10 | A/G | 0.017 | LTBP1 | 1.25E-06 |
| FI-Y12 | 4 | 24.54-24.58 | 1 | 24557163 | 12 | A/G | 0.013 |  | 1.75E-06 |
| FI-Y12 | 4 | 37.89-37.93 | 1 | 37912326 | 10 | T/C | 0.021 | OSR2/RF00100 | 2.37E-06 |
| FI-Y12 | 6 | 11.85-11.89 | 1 | 11870758 | 21 | A/T | 0.138 |  | 2.89E-06 |
| FI-Y12 | 6 | 13.75-13.79 | 2 | 13765218 | 13 | T/C | 0.032 | VAC14 | 4.52E-07 |
| FI-Y12 | 6 | 15.33-15.37 | 1 | 15350761 | 1 | A/G | 0.028 |  | 3.02E-06 |
| FI-Y12 | 6 | 16.10-16.14 | 1 | 16124138 | 1 | G/C | 0.026 |  | 8.18E-07 |
| FI-Y12 | 6 | 18.65-18.69 | 1 | 18671026 | 20 | C/T | 0.037 | MT1D/LOC100739663/MT1A | 3.31E-06 |
| FI-Y12 | 6 | 27.78-27.82 | 1 | 27804515 | 0 | C/T | 0.024 | C16orf70/B3GNT9/TRADD/HSF4/NOL3/KIAA0895L | 2.54E-07 |
| FI-Y12 | 7 | 2.04-2.08 | 7 | 2061575 | 0 | C/T | 0.018 | SLC22A23 | 1.22E-06 |
| FI-Y12 | 7 | 2.08-2.12 | 1 | 2101679 | 0 | G/A | 0.018 |  | 1.22E-06 |
| FI-Y12 | 7 | 2.12-2.16 | 1 | 2138975 | 0 | G/A | 0.02 |  | 4.15E-06 |
| FI-Y12 | 7 | 2.14-2.18 | 2 | 2161490 | 3 | C/T | 0.026 |  | 2.95E-07 |
| FI-Y12 | 7 | 2.20-2.24 | 3 | 2221001 | 0 | G/A | 0.018 | PXDC1 | 1.22E-06 |
| FI-Y12 | 7 | 2.23-2.27 | 5 | 2252385 | 2 | G/A | 0.018 |  | 1.17E-06 |
| FI-Y12 | 7 | 2.78-2.82 | 1 | 2801404 | 4 | T/G | 0.018 |  | 1.25E-06 |
| FI-Y12 | 7 | 2.93-2.97 | 3 | 2946416 | 8 | C/A | 0.017 | CDYL | 2.68E-07 |
| FI-Y12 | 7 | 3.08-3.12 | 1 | 3103789 | 13 | C/T | 0.017 | PPP1R3G/LYRM4 | 5.88E-07 |
| FI-Y12 | 7 | 6.68-6.72 | 1 | 6699509 | 3 | T/C | 0.022 |  | 1.27E-06 |
| FI-Y12 | 7 | 6.85-6.89 | 1 | 6865076 | 2 | A/G | 0.022 |  | 6.67E-07 |
| FI-Y12 | 7 | 7.00-7.04 | 1 | 7023787 | 19 | A/G | 0.022 |  | 2.44E-07 |
| FI-Y12 | 7 | 7.11-7.15 | 1 | 7132671 | 0 | T/C | 0.022 |  | 1.18E-06 |
| FI-Y12 | 7 | 126.45-126.49 | 1 | 126470008 | 0 | A/C | 0.018 |  | 9.62E-07 |
| FI-Y12 | 10 | 3.76-3.80 | 1 | 3781065 | 6 | C/T | 0.033 |  | 1.99E-06 |
| FI-Y12 | 10 | 3.71-3.75 | 1 | 3733125 | 20 | A/T | 0.031 |  | 3.34E-06 |
| FI-Y12 | 10 | 23.50-23.54 | 1 | 23516436 | 12 | A/T | 0.025 | CACNA1S | 3.55E-06 |
| FI-Y12 | 11 | 79.47-79.51 | 1 | 79491465 | 5 | T/C | 0.016 |  | 2.58E-06 |
| FI-Y12 | 12 | 17.18-17.22 | 1 | 17195831 | 18 | G/C | 0.022 | MAPT | 3.05E-06 |
| FI-Y12 | 12 | 19.29-19.33 | 1 | 19311388 | 1 | T/C | 0.018 | MPP3/CFAP97D1 | 3.65E-06 |
| FI-Y12 | 12 | 24.15-24.19 | 1 | 24166383 | 13 | G/T | 0.015 | SP2 | 1.45E-06 |
| FI-Y12 | 12 | 35.57-35.61 | 4 | 35585246 | 18 | G/A | 0.019 | YPEL2 | 1.91E-06 |
| FI-Y12 | 12 | 48.03-48.07 | 1 | 48047432 | 0 | G/A | 0.016 | RPA1 | 2.63E-07 |
| FI-Y12 | 12 | 59.16-59.20 | 1 | 59175870 | 14 | G/T | 0.013 | UBB | 8.82E-07 |
| FI-Y12 | 13 | 163.57-163.61 | 1 | 163586325 | 12 | G/A | 0.013 |  | 1.18E-06 |
| FI-Y12 | 14 | 10.69-10.73 | 1 | 10709779 | 17 | C/T | 0.013 | ADRA1A | 1.30E-06 |
| FI-Y12 | 14 | 15.19-15.23 | 1 | 15211795 | 0 | A/T | 0.028 |  | 1.38E-06 |
| FI-Y12 | 14 | 37.13-37.17 | 1 | 37154712 | 2 | A/G | 0.022 |  | 9.56E-07 |
| FI-Y12 | 14 | 151.01-151.05 | 2 | 151031781 | 0 | C/T | 0.032 |  | 1.20E-06 |
| FI-Y12 | 14 | 151.11-151.15 | 1 | 151129973 | 3 | A/G | 0.085 |  | 2.26E-06 |
| FI-Y12 | 14 | 151.43-151.47 | 1 | 151452098 | 0 | G/C | 0.094 |  | 3.84E-06 |
| FI-Y12 | 14 | 151.47-151.51 | 3 | 151491845 | 3 | C/G | 0.1 |  | 1.83E-06 |
| FI-Y12 | 14 | 151.51-151.55 | 1 | 151526498 | 0 | C/A | 0.094 |  | 3.84E-06 |
| FI-Y12 | 15 | 4.09-4.13 | 1 | 4110217 | 10 | A/G | 0.021 | ORC4 | 1.34E-06 |
| FI-Y12 | 15 | 4.19-4.23 | 1 | 4214327 | 0 | A/G | 0.024 | ACVR2A | 3.21E-06 |
| FI-Y12 | 15 | 4.38-4.42 | 3 | 4396946 | 0 | G/A | 0.022 |  | 9.06E-07 |
| FI-Y12 | 15 | 4.43-4.47 | 3 | 4449450 | 16 | G/A | 0.021 |  | 2.72E-07 |
| FI-Y12 | 15 | 4.67-4.71 | 3 | 4694130 | 5 | C/T | 0.023 |  | 8.77E-07 |
| FI-Y12 | 15 | 4.99-5.03 | 1 | 5014047 | 16 | A/C | 0.026 |  | 2.80E-06 |
| FI-Y12 | 15 | 5.46-5.50 | 1 | 5484163 | 0 | A/G | 0.024 |  | 4.49E-07 |
| FI-Y12 | 15 | 5.70-5.74 | 1 | 5718286 | 1 | C/G | 0.024 |  | 4.15E-07 |
| FI-Y12 | 15 | 18.70-18.74 | 1 | 18716962 | 15 | T/A | 0.013 |  | 2.37E-06 |
| FI-Y12 | 17 | 11.24-11.28 | 1 | 11260626 | 15 | G/A | 0.053 |  | 2.88E-06 |
| FI-Y12 | 17 | 12.05-12.09 | 1 | 12067529 | 16 | T/C | 0.013 | INTS10 | 1.58E-06 |
| FI-Y12 | 17 | 15.62-15.66 | 1 | 15637209 | 6 | A/G | 0.019 |  | 8.16E-07 |
| FI-Y12 | 17 | 16.51-16.55 | 2 | 16528184 | 1 | A/G | 0.018 |  | 2.71E-07 |
| FI-Y12 | 17 | 33.39-33.43 | 1 | 33407842 | 20 | T/G | 0.022 | STK35 | 1.85E-06 |
| FI-Y12 | 17 | 33.43-33.47 | 2 | 33447145 | 16 | C/G | 0.021 |  | 2.19E-06 |
| FI-Y12 | 17 | 62.51-62.55 | 2 | 62529371 | 11 | C/T | 0.044 | EEF1A2/PTK6/SRMS | 3.14E-07 |
| FI-Y23 | 1 | 144.43-144.47 | 1 | 144445871 | 9 | T/C | 0.013 | FAM189A1 | 2.83E-07 |
| FI-Y23 | 1 | 145.83-145.87 | 1 | 145852999 | 13 | G/T | 0.015 |  | 2.86E-07 |
| FI-Y23 | 1 | 292.85-292.89 | 1 | 292867707 | 9 | G/A | 0.026 |  | 9.98E-07 |
| FI-Y23 | 1 | 28.53-28.57 | 1 | 28546905 | 5 | C/A | 0.013 | AHI1 | 3.62E-06 |
| FI-Y23 | 2 | 9.16-9.20 | 1 | 9182321 | 21 | C/A | 0.011 | EEF1G/AHNAK | 5.62E-07 |
| FI-Y23 | 3 | 51.20-51.24 | 1 | 51220936 | 17 | G/T | 0.02 |  | 6.93E-07 |
| FI-Y23 | 3 | 50.17-50.21 | 1 | 50189811 | 7 | A/C | 0.013 |  | 9.65E-07 |
| FI-Y23 | 5 | 70.22-70.26 | 1 | 70235844 | 16 | G/A | 0.081 | USP18 | 2.20E-06 |
| FI-Y23 | 5 | 96.97-97.01 | 1 | 96994782 | 12 | A/G | 0.013 | LRRIQ1/TSPAN19 | 2.95E-06 |
| FI-Y23 | 5 | 69.78-69.82 | 1 | 69800963 | 3 | A/G | 0.083 | BCL2L13 | 3.95E-06 |
| FI-Y23 | 5 | 39.01-39.05 | 1 | 39033093 | 9 | T/C | 0.023 |  | 4.21E-06 |
| FI-Y23 | 5 | 70.82-70.86 | 1 | 70842638 | 4 | C/G | 0.077 |  | 4.52E-06 |
| FI-Y23 | 6 | 126.71-126.75 | 1 | 126732428 | 9 | C/A | 0.017 |  | 6.27E-07 |
| FI-Y23 | 6 | 62.53-62.57 | 1 | 62554896 | 5 | G/T | 0.017 | ZNF671/LOC100737912 | 1.71E-06 |
| FI-Y23 | 7 | 21.69-21.73 | 1 | 21706036 | 12 | A/G | 0.011 | OR2B6/LOC100512238 | 4.14E-07 |
| FI-Y23 | 8 | 118.33-118.37 | 1 | 118349765 | 9 | C/A | 0.028 | MANBA | 1.20E-06 |
| FI-Y23 | 9 | 130.10-130.14 | 1 | 130123875 | 3 | C/A | 0.015 |  | 4.43E-07 |
| FI-Y23 | 12 | 19.98-20.02 | 1 | 19998592 | 9 | C/A | 0.023 | LOC110256000/LOC100520329 | 1.65E-06 |
| FI-Y23 | 14 | 69.62-69.66 | 1 | 69643308 | 18 | G/T | 0.011 | CTNNA3 | 3.47E-07 |
| FI-Y23 | 14 | 64.93-64.97 | 1 | 64949599 | 12 | A/G | 0.011 |  | 1.65E-06 |
| FI-Y23 | 16 | 6.64-6.68 | 1 | 6659242 | 3 | T/A | 0.025 |  | 3.20E-06 |
| FI-Y23 | 18 | 11.86-11.90 | 1 | 11882910 | 18 | G/T | 0.013 | DGKI | 8.24E-07 |
| FI-Y34 | 1 | 143.82-143.86 | 1 | 143843202 | 0 | T/C | 0.022 | TRPM1 | 3.26E-07 |
| FI-Y34 | 1 | 287.21-287.25 | 1 | 287226577 | 15 | T/G | 0.013 |  | 1.08E-06 |
| FI-Y34 | 1 | 8.28-8.32 | 1 | 8303571 | 18 | G/T | 0.037 |  | 4.12E-06 |
| FI-Y34 | 2 | 51.78-51.82 | 1 | 51801986 | 0 | T/C | 0.041 |  | 3.58E-07 |
| FI-Y34 | 2 | 54.73-54.77 | 1 | 54754687 | 14 | G/T | 0.015 |  | 9.13E-07 |
| FI-Y34 | 2 | 138.82-138.86 | 2 | 138838025 | 17 | T/A | 0.304 |  | 4.78E-06 |
| FI-Y34 | 4 | 53.63-53.67 | 1 | 53650599 | 3 | A/T | 0.466 |  | 7.16E-07 |
| FI-Y34 | 6 | 66.62-66.66 | 1 | 66640078 | 12 | G/A | 0.015 |  | 4.90E-07 |
| FI-Y34 | 9 | 38.19-38.23 | 5 | 38206875 | 10 | C/T | 0.058 | ZC3H12C/RDX | 5.06E-07 |
| FI-Y34 | 9 | 41.07-41.11 | 9 | 41085054 | 6 | T/C | 0.082 | DRD2 | 2.52E-06 |
| FI-Y34 | 9 | 109.59-109.63 | 1 | 109607424 | 3 | T/G | 0.015 |  | 1.10E-06 |
| FI-Y34 | 10 | 8.06-8.10 | 2 | 8075830 | 18 | C/G | 0.024 | SPATA17 | 1.47E-06 |
| FI-Y34 | 11 | 52.69-52.73 | 1 | 52705197 | 0 | T/C | 0.012 |  | 1.83E-06 |
| FI-Y34 | 11 | 53.29-53.33 | 2 | 53310491 | 1 | G/C | 0.024 |  | 2.84E-06 |
| FI-Y34 | 12 | 43.86-43.90 | 1 | 43876030 | 18 | T/C | 0.066 | WSB1 | 2.68E-06 |
| FI-Y34 | 13 | 39.27-39.31 | 7 | 39293665 | 10 | G/A | 0.018 |  | 2.63E-06 |
| FI-Y34 | 14 | 72.15-72.19 | 1 | 72171837 | 13 | T/G | 0.018 | SRGN | 3.70E-06 |
| FI-Y34 | 15 | 154.55-154.59 | 2 | 154566616 | 0 | G/A | 0.139 |  | 7.04E-07 |
| FI-Y34 | 15 | 140.92-140.96 | 1 | 140939604 | 14 | T/A | 0.023 |  | 1.14E-06 |
| FI-Y34 | 15 | 157.56-157.60 | 1 | 157579834 | 3 | C/T | 0.144 |  | 1.28E-06 |
| FI-Y34 | 15 | 153.85-153.89 | 1 | 153869922 | 1 | C/A | 0.14 |  | 4.26E-06 |
| FI-Y34 | 17 | 54.30-54.34 | 1 | 54319915 | 7 | A/G | 0.022 |  | 1.27E-06 |
| FI-Y34 | 18 | 22.67-22.71 | 1 | 22689096 | 17 | C/T | 0.016 |  | 1.55E-06 |

Supplementary Table 3. The suggestive SNPs using imputed WGS data (allelic R^2^ > 0.3) for FI traits of different parities in Landrace and Large White pigs.

| Traits | Chromosome | Range of SNP (Mb) | Number of SNP | Top SNP Position (bp) | n_miss | Allele | Allele Frequency | Candidate gene | P-value |
| --- | --- | --- | --- | --- | --- | --- | --- | --- | --- |
| FI-L12 | 1 | 12.76-12.80 | 1 | 12783356 | 0 | G/A | 0.013 |  | 8.42E-08 |
| FI-L23 | 6 | 77.40-77.44 | 6 | 77416585 | 0 | T/C | 0.031 | ALDH4A1/IFFO2 | 2.41E-08 |
| FI-L34 | 4 | 91.47-91.51 | 7 | 91492978 | 0 | A/G | 0.037 | OR6N2/OR6N1/OR6K6/LOC110260312 | 6.98E-08 |
| FI-L34 | **5** | **79.99-80.03** | **1** | **80012051** | **0** | **C/T** | **0.017** | **CHST11** | **2.13E-07** |
| FI-Y12 | 3 | 136.58-136.62 | 25 | 136602890 | 0 | C/T | 0.014 |  | 9.62E-08 |
| FI-Y12 | 6 | 27.78-27.82 | 5 | 27804549 | 0 | A/G | 0.022 | C16orf70/B3GNT9/TRADD/HSF4/NOL3/KIAA0895L | 2.83E-08 |
| FI-Y12 | 7 | 2.14-2.18 | 2 | 2158189 | 0 | G/C | 0.024 |  | 1.72E-08 |
| FI-Y12 | 14 | 10.67-10.71 | 5 | 10689064 | 0 | A/G | 0.058 | ADRA1A | 3.02E-08 |
| FI-Y12 | 14 | 11.63-11.67 | 1 | 11650048 | 0 | T/C | 0.114 | SCARA5 | 1.10E-07 |
| FI-Y12 | 14 | 37.18-37.22 | 5 | 37197399 | 0 | A/G | 0.02 |  | 1.36E-07 |
| FI-Y12 | 15 | 4.90-4.94 | 1 | 4919126 | 0 | A/C | 0.022 |  | 6.28E-08 |
| FI-Y12 | 17 | 63.20-63.24 | 5 | 63224611 | 0 | T/G | 0.054 |  | 3.87E-08 |
| FI-Y12 | 17 | 15.62-15.66 | 5 | 15635902 | 0 | G/A | 0.016 |  | 4.47E-08 |
| FI-Y12 | 17 | 11.19-11.23 | 9 | 11214141 | 0 | C/T | 0.046 | PLAT | 1.04E-07 |
| FI-Y12 | 17 | 16.52-16.56 | 17 | 16543976 | 0 | G/T | 0.016 |  | 4.47E-08 |
| FI-Y12 | 17 | 16.53-16.57 | 31 | 16553663 | 0 | C/G | 0.016 |  | 4.47E-08 |
| FI-Y23 | 3 | 120.16-120.20 | 1 | 120181013 | 0 | C/T | 0.023 | SMC6/VSNL1 | 7.67E-08 |
| **FI-Y34** | **9** | **38.20-38.24** | **119** | **38215712** | **0** | **G/A** | **0.067** | **ZC3H12C/RDX** | **3.74E-08** |
| FI-Y34 | 15 | 154.78-154.82 | 5 | 154798586 | 0 | C/A | 0.156 |  | 1.59E-08 |
| FI-Y34 | 15 | 154.91-154.95 | 2 | 154925066 | 0 | C/T | 0.26 |  | 5.88E-08 |
| FI-Y34 | 15 | 154.92-154.96 | 6 | 154942807 | 0 | T/C | 0.224 |  | 1.02E-08 |

Supplementary Table 4. The suggestive SNPs using imputed WGS data (allelic R^2^ > 0.8) for FI traits of different parities in Landrace and Large White pigs.

| Trait | Chromosome | Range of SNP (Mb) | Number of SNP | Top SNP Position (bp) | n_miss | Allele | Allele Frequency | Candidate gene | P-value |
| --- | --- | --- | --- | --- | --- | --- | --- | --- | --- |
| FI-L12 | 1 | 12.76-12.80 | 1 | 12783356 | 0 | G/A | 0.013 |  | 1.20E-06 |
| FI-Y12 | 1 | 155.40-155.44 | 1 | **155417340** | 24 | G/T | 0.013 |  | 3.38E-07 |
| FI-Y12 | 6 | 27.78-27.82 | 6 | **27804549** | 0 | A/G | 0.022 | C16orf70/B3GNT9/TRADD/HSF4/NOL3/KIAA0895L | 1.69E-07 |
| FI-Y12 | 12 | 48.03-48.07 | 1 | **48047432** | 0 | G/A | 0.016 | RPA1 | 1.02E-06 |
| FI-Y12 | 14 | 37.18-37.22 | 3 | **37197399** | 0 | A/G | 0.02 |  | 5.96E-07 |
| FI-Y12 | 17 | 63.20-63.24 | 3 | **63224611** | 0 | T/G | 0.054 |  | 2.18E-08 |
| FI-Y12 | 17 | 16.52-16.56 | 28 | **16543976** | 0 | G/T | 0.016 |  | 4.47E-08 |
| FI-Y12 | 17 | 16.53-16.57 | 23 | **16553663** | 0 | C/G | 0.016 |  | 4.47E-08 |
| FI-Y34 | 9 | 38.20-38.24 | 73 | **38215712** | 0 | G/A | 0.067 | ZC3H12C/RDX | 3.75E-08 |

Supplementary Table 5. The genomic inflation factor (λ) for each GWAS.

| Trait | λ (GBS data) | Imputed WGS data | |
| --- | --- | --- | --- |
|  |  | λ (Filtering R^2^>0.3) | λ (Filtering R^2^>0.8) |
| FI_L12 | 1.05 | 1.03 | 1.03 |
| FI_L23 | 0.99 | 0.97 | 0.95 |
| FI_L34 | 1.00 | 0.95 | 0.94 |
| FI_Y12 | 1.17 | 1.02 | 0.99 |
| FI_Y23 | 1.08 | 1.01 | 1.02 |
| FI_Y34 | 1.08 | 1.03 | 1.03 |


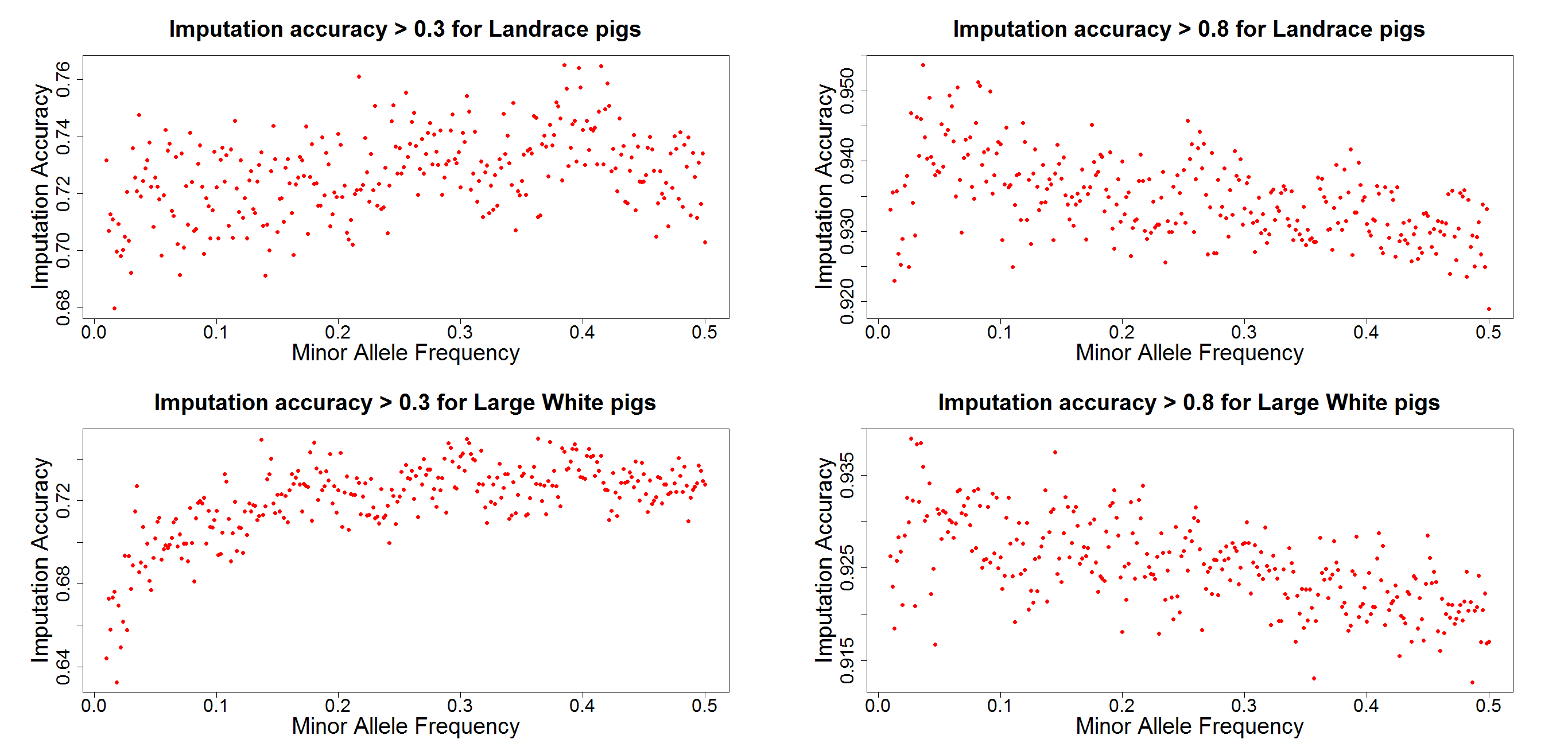


Supplementary Figure 1. Average imputation accuracies versus minor allele frequency based on allelic R^2^ >0.3 (left) and R^2^ >0.8 (right) for Landrace and Large White pigs. The SNPs were classified by minor allele frequency.


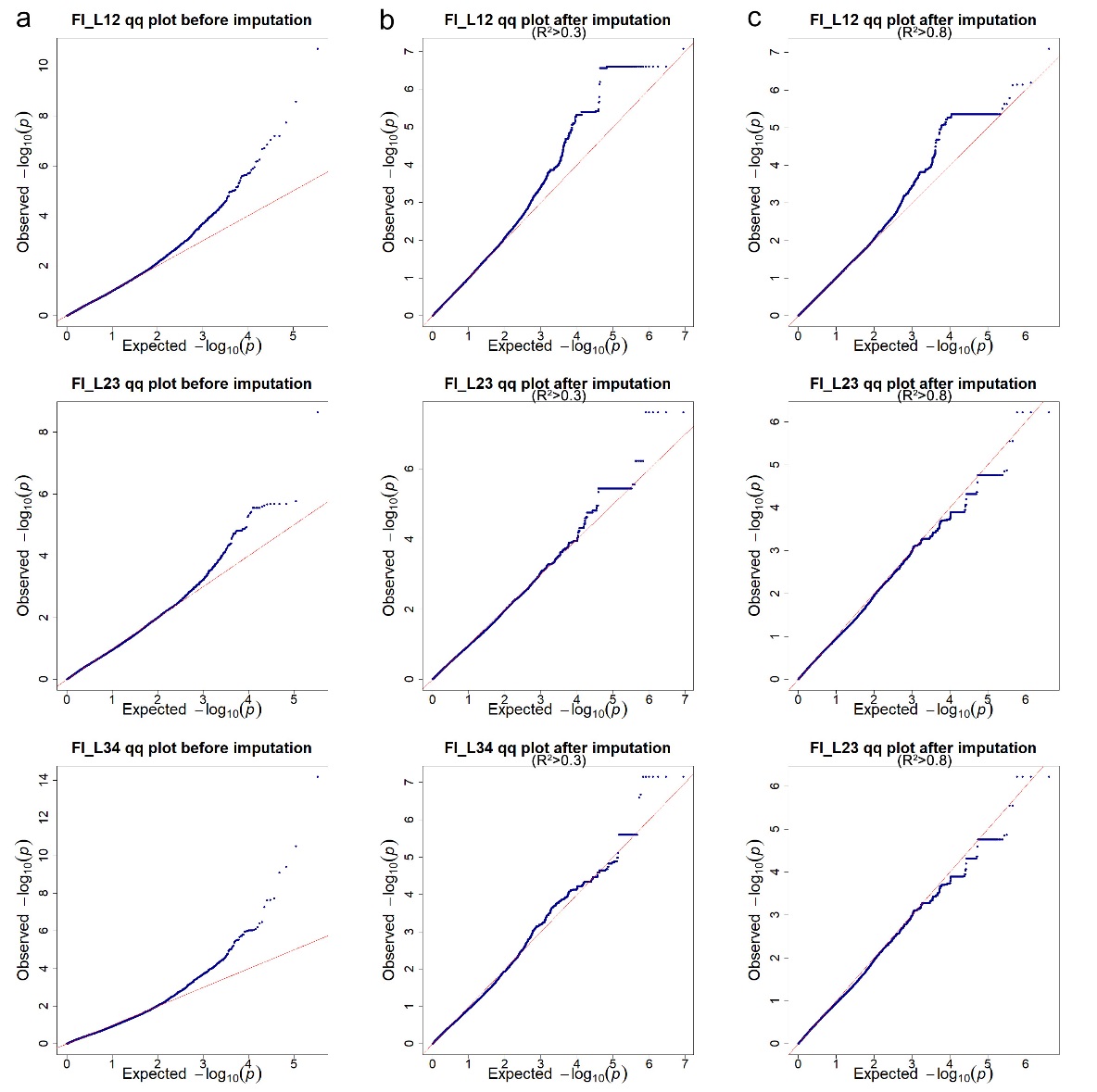


Supplementary Figure 2. The QQ-plot association results for FI trait of different parities using different SNP data (a GBS data, b imputed WGS data with allelic R2 > 0.3, c imputed WGS data with allelic R2 > 0.8) in Landrace pigs.


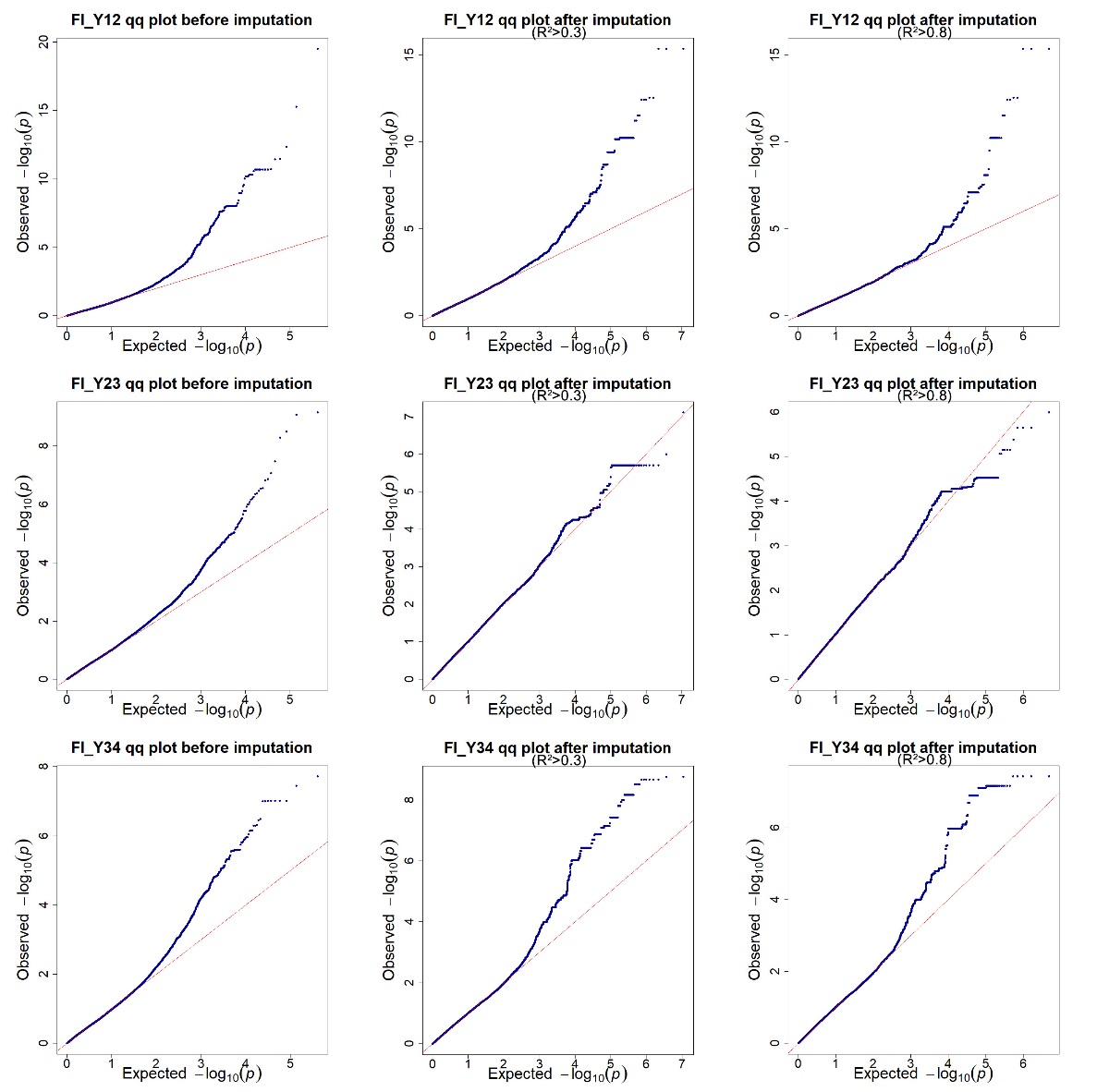


Supplementary Figure 3. The QQ-plot association results for FI trait of different parities using different SNP data (a GBS data, b imputed WGS data with allelic R2 > 0.3, c imputed WGS data with allelic R2 > 0.8) in Large White pigs.


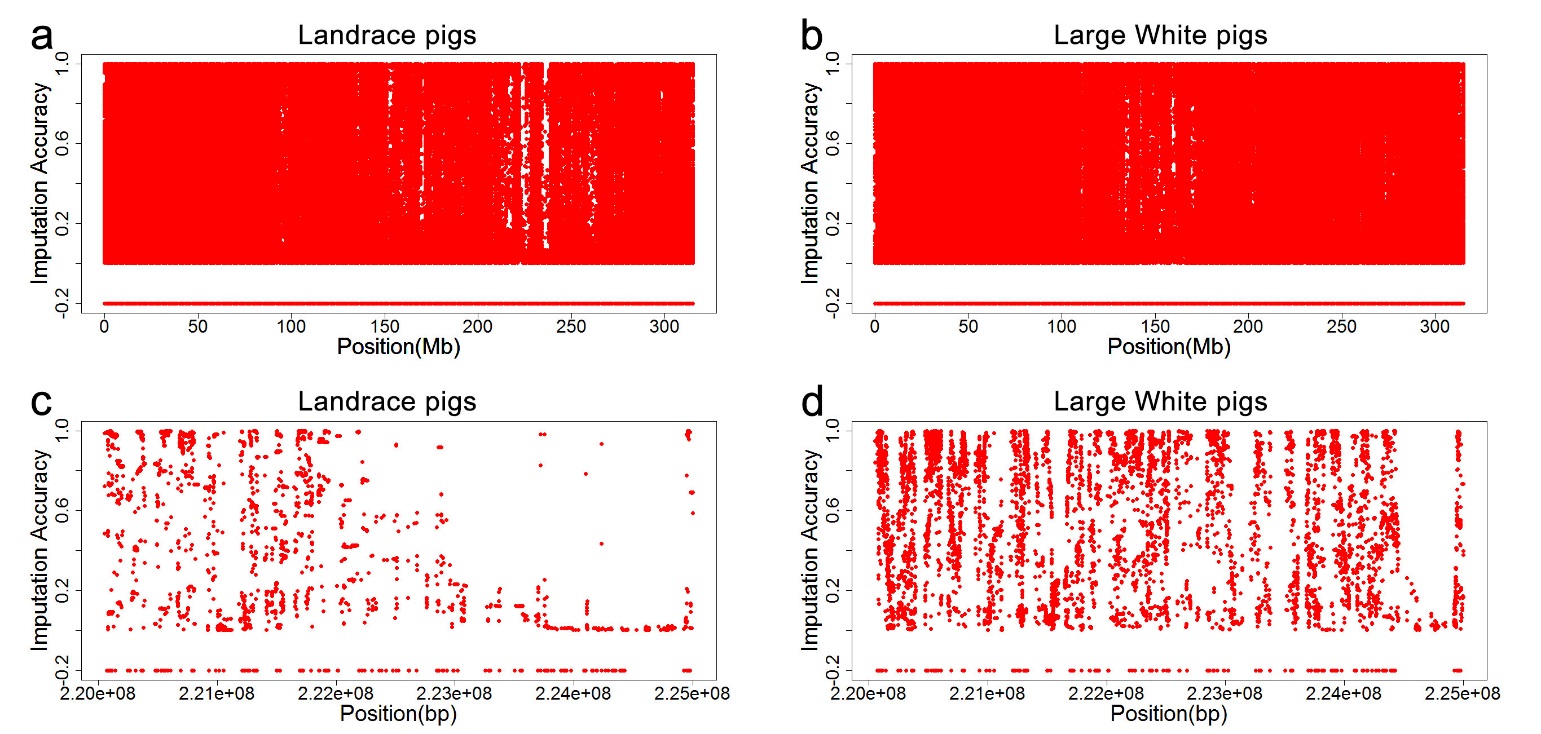


Supplementary Figure 4. The imputation accuracy on chromosome 1 for two populations (left Landrace pigs, right Large White pigs). The distribution of imputation accuracy versus the location on chromosome 1 (a and b) for the whole chromosome and (c and d) for the region between 220 and 225 Mb on chromosome 1. The Line at -0.2 indicates the position of GBS data on this chromosome.


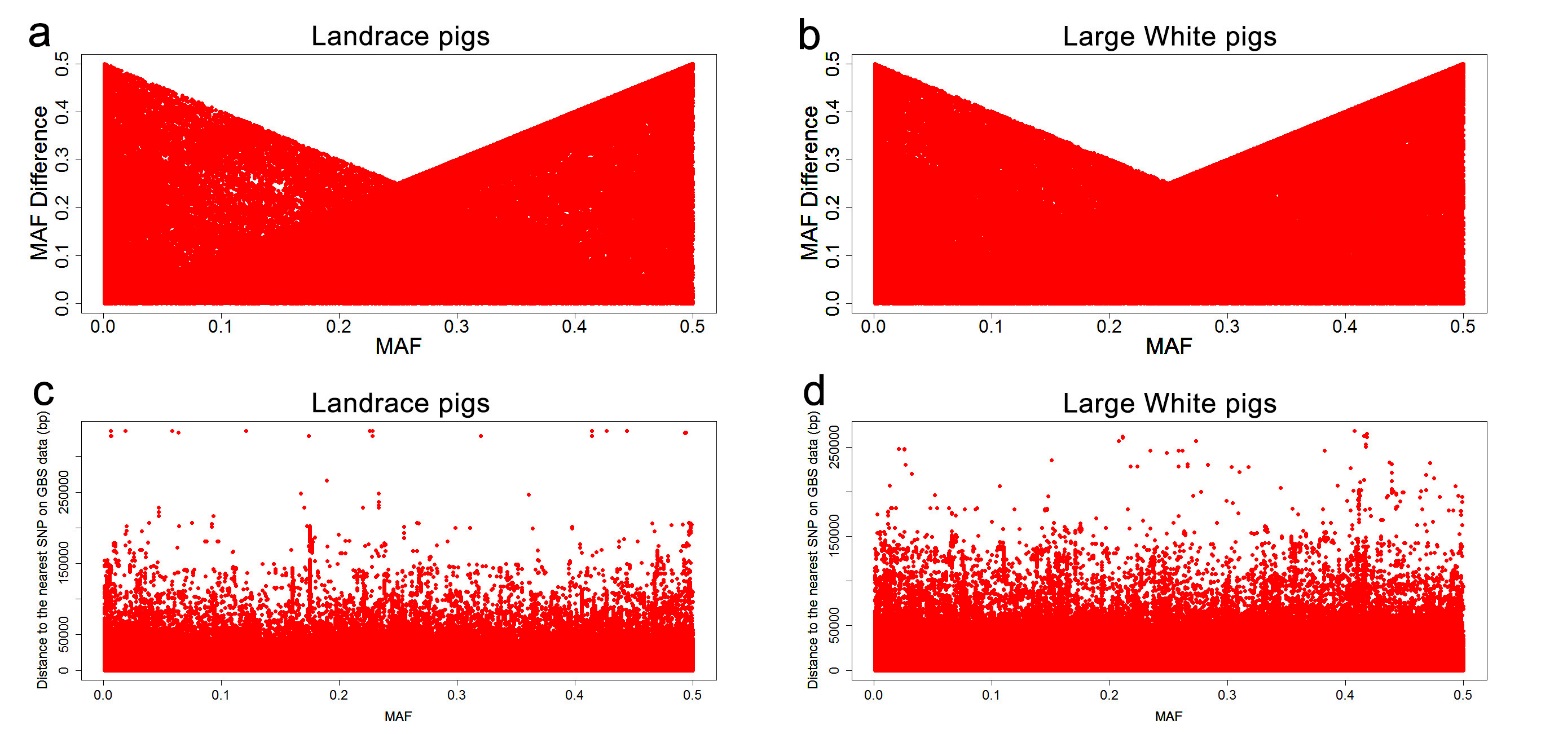


Supplementary Figure 5. The distance and MAF difference between imputed SNPs and their nearest SNPs on GBS data versus MAF of imputed SNPs in two populations.
